# Regulation of Wnt/PCP signaling through p97/VCP-KBTBD7–mediated Vangl ubiquitination and endoplasmic reticulum–associated degradation

**DOI:** 10.1126/sciadv.abg2099

**Published:** 2021-05-14

**Authors:** Di Feng, Jin Wang, Wei Yang, Jingyu Li, Xiaochen Lin, Fangzi Zha, Xiaolu Wang, Luyao Ma, Nga Ting Choi, Yusuke Mii, Shinji Takada, Michael S. Y. Huen, Yusong Guo, Liang Zhang, Bo Gao

**Affiliations:** 1 School of Biomedical Sciences, Li Ka Shing Faculty of Medicine, The University of Hong Kong, Pokfulam, Hong Kong SAR, China.; 2 The University of Hong Kong-Shenzhen Institute of Research and Innovation (HKU-SIRI), Shenzhen, China.; 3 Department of Biomedical Sciences, College of Veterinary Medicine and Life Sciences, City University of Hong Kong, Hong Kong SAR, China.; 4 Exploratory Research Center on Life and Living Systems (ExCELLS) and National Institute for Basic Biology, National Institutes of Natural Sciences, Okazaki, Japan.; 5 Japan Science and Technology Agency, PRESTO, Kawaguchi, Japan.; 6 Division of Life Science, Hong Kong University of Science and Technology, Hong Kong SAR, China.

## Abstract

The four-pass transmembrane proteins Vangl1 and Vangl2 are dedicated core components of Wnt/planar cell polarity (Wnt/PCP) signaling that critically regulate polarized cell behaviors in many morphological and physiological processes. Here, we found that the abundance of Vangl proteins is tightly controlled by the ubiquitin-proteasome system through endoplasmic reticulum–associated degradation (ERAD). The key ERAD component p97/VCP directly binds to Vangl at a highly conserved VCP-interacting motif and recruits the E3 ligase KBTBD7 via its UBA-UBX adaptors to promote Vangl ubiquitination and ERAD. We found that Wnt5a/CK1 prevents Vangl ubiquitination and ERAD by inducing Vangl phosphorylation, which facilitates Vangl export from the ER to the plasma membrane. We also provide in vivo evidence that KBTBD7 regulates convergent extension during zebrafish gastrulation and functions as a tumor suppressor in breast cancer by promoting Vangl degradation. Our findings reveal a previously unknown regulatory mechanism of Wnt/PCP signaling through the p97/VCP-KBTBD7–mediated ERAD pathway.

## Teaser

The abundance of core Wnt/PCP protein Vangl is regulated by the endoplasmic reticulum–associated degradation (ERAD) pathway.

## INTRODUCTION

Wnt signaling is an evolutionarily conserved cellular mechanism that regulates nearly every aspect of development and homeostasis in both physiological and pathological conditions (*1*). As one of the three major Wnt pathways [Wnt/ß-catenin, Wnt/planar cell polarity (PCP), and Wnt/calcium], Wnt/PCP signaling conveys directional information to control polarized cell behavior, a process involving the asymmetric segregation of a set of core proteins within the cell and the coordinated cell polarization across the tissue plane (*2*, *3*). PCP has been extensively studied in both invertebrates and vertebrates. Genetic studies in *drosophila* identified a group of core PCP proteins: Frizzled (Fz), Van Gogh (Vang), Dishevelled (Dsh), Prickle (Pk), Diego (Dgo), and Flamingo (Fmi), which control the orientation of bristles and hairs on the body and the arrangement of ommatidia in the compound eye (*4*). In vertebrates such as zebrafish, PCP is required for a process known as convergent extension (CE) movement, which contributes to the elongation of their anterior-posterior (A-P) body axis (*5*). In mammals, PCP has been shown to control a diverse array of cellular, developmental, and physiological processes (*6*, *7*). Disruption of PCP underlies a variety of human developmental defects, whereas activation of PCP has been implicated in the progression and metastasis of various cancers (*8*, *9*). This pathway is particularly sensitive to the levels of the core PCP proteins, as the establishment of PCP asymmetry depends on both the intracellular and intercellular interactions of these proteins. Overexpression or loss of any of the core proteins is generally sufficient to randomize the polarity of the others, resulting in PCP defects. An understanding of how PCP signaling is transduced and regulated is therefore essential to harness this signaling pathway for therapeutic purposes. Although the requirement of Wnt ligands for PCP in *drosophila* is controversial (*10*–*12*), it is well accepted that Wnt ligands (particularly Wnt5a and Wnt11) are required for PCP in vertebrates (*13*–*20*), the so-called Wnt/PCP signaling pathway (*2*, *3*, *21*).

The tetraspan membrane protein Vang (*Van Gogh*, also known as *strabismus*/*stbm*) is one of core PCP proteins originally identified in *drosophila*. Either overexpression or inactivation of *Vang* results in the randomized orientation of bristles and hairs (*22*–*24*). *Vang-like 1* (*Vangl1*) and *Vang-like 2* (*Vangl2*) are the two vertebrate homologs of *drosophila Vang*. Similar to *drosophila Vang*, either overexpression or loss of function of *Vangl2* in zebrafish causes impaired CE movement, a typical Wnt/PCP defect (*25*, *26*). In mammals, *Vangl1* and *Vangl2* are redundantly required for PCP in a dose-dependent manner, with *Vangl2* being genetically more important than *Vangl1* during embryonic development (*27*, *28*). *Vangl* mutant mice exhibited severe defects in numerous tissues and organs, which highlights the broad and important roles of *Vangl* in mammalian development (*29*). Missense mutations in *Vangl* have been reported in patients with neural tube defects (*30*, *31*), and aberrant Vangl activation has been strongly implicated in breast cancer and rhabdomyosarcoma (*32*–*35*). However, despite the importance of Vangl in development and diseases, the molecular mechanisms underlying the control of Vangl protein quality and quantity remain poorly understood.

The endoplasmic reticulum (ER) is the major site of membrane protein folding, assembly, and maturation. Endoplasmic reticulum–associated degradation (ERAD) is a conserved quality control mechanism that removes misfolded or unassembled proteins, which ensures only properly folded and assembled proteins are transported to their final destinations. ERAD substrates are ubiquitinated and then extracted from the ER membrane to the cytosol, where they are degraded by proteasomes. This process requires ER membrane–embedded protein complex, such as Hrd1 and Doa10/March6, and the ubiquitin-dependent segregase VCP (valosin-containing protein, also known as p97) (*36*–*38*). The ERAD process is critical for cell homeostasis by regulating the protein levels of many signaling molecules especially those localized to the plasma membrane under physiological conditions. The involvement of ERAD in canonical Wnt/b-catenin signaling has been recently reported (*39*, *40*), but whether ERAD contributes to Wnt/PCP signaling or to the homeostatic regulation of the core PCP proteins is unknown.

Ubiquitin-mediated regulatory mechanisms have been found to play important roles in modulating Wnt/PCP signaling via its core proteins such as Dsh, Pk, or Fmi (*41*–*45*). For instance, Dsh or its vertebrate homologs can be targeted by multiple E3 ubiquitin ligases, including Cul3-Dbo-Kelch, Cul3-KLHL12, NEDD4L, PDZRN3, or NRDP1, which affect Wnt/PCP signaling in different contexts (*42*, *43*, *45*–*47*). Similarly, Pk is regulated by ubiquitination through Cul1-SkpA-Slimb or Smurf1/2 HECT E3 ligases in *drosophila* and mammalian cells, respectively (*41*, *44*). It was also found that a deubiquitinating enzyme Faf stabilizes Fmi, which is important for PCP (*42*). However, the ubiquitin-mediated regulation of Vang/Vangl has not been reported. The process of ubiquitination consists of several sequential steps involving a cascade of enzymes including E1, E2, and E3 ubiquitin ligases. The substrate specificity in ubiquitination is mainly conferred by more than 600 E3 ubiquitin ligases, of which Cullin (CUL)–RING ubiquitin ligases (CRLs) represent the largest family (*48*). In the CRL complex, the CUL-RBX module constitutes the catalytic core, and the substrate recognition proteins define the specificity. For example, the Cullin 3 (CUL3) substrate recognition Broad-Complex, tramtrack and bric à brac (BTB)-Kelch proteins specifically bind to CUL3 through the BTB domain but recruit substrates through the Kelch domain (*49*).

Here, we report that CUL3-KBTBD7 (Kelch repeat and BTB domain containing 7) ubiquitin ligase complex targets and degrades Vangl via the ERAD pathway. Unexpectedly, we identified a p97/VCP-interacting motif (VIM) on Vangl, which is required for Vangl-p97/VCP direct interaction and KBTBD7-induced Vangl ubiquitination and degradation. The p97/VCP recruits KBTBD7 to Vangl via its UBA-UBX (ubiquitin-associated and ubiquitin-like) adaptors (SAKS1, UBXD7, and FAF1). These findings imply a new mechanism of action of p97/VCP in regulating its substrates. Furthermore, we found that Wnt5a inhibits Vangl ubiquitination and ERAD by inducing Vangl phosphorylation in the ER and promoting its transportation to the cell surface. In zebrafish, KBTBD7 expression resulted in Vangl degradation and CE defects. Degradation of Vangl proteins by KBTBD7 also led to reduced breast cancer growth and metastasis in mouse xenograft models. Our studies reveal a negative role of KBTBD7 in Wnt/PCP signaling and suggest that the p97/VCP-mediated ERAD pathway is important in the control of the quality and quantity of PCP proteins.

## RESULTS

### ERAD and ubiquitination of Vangl proteins

**Fig. 1:**
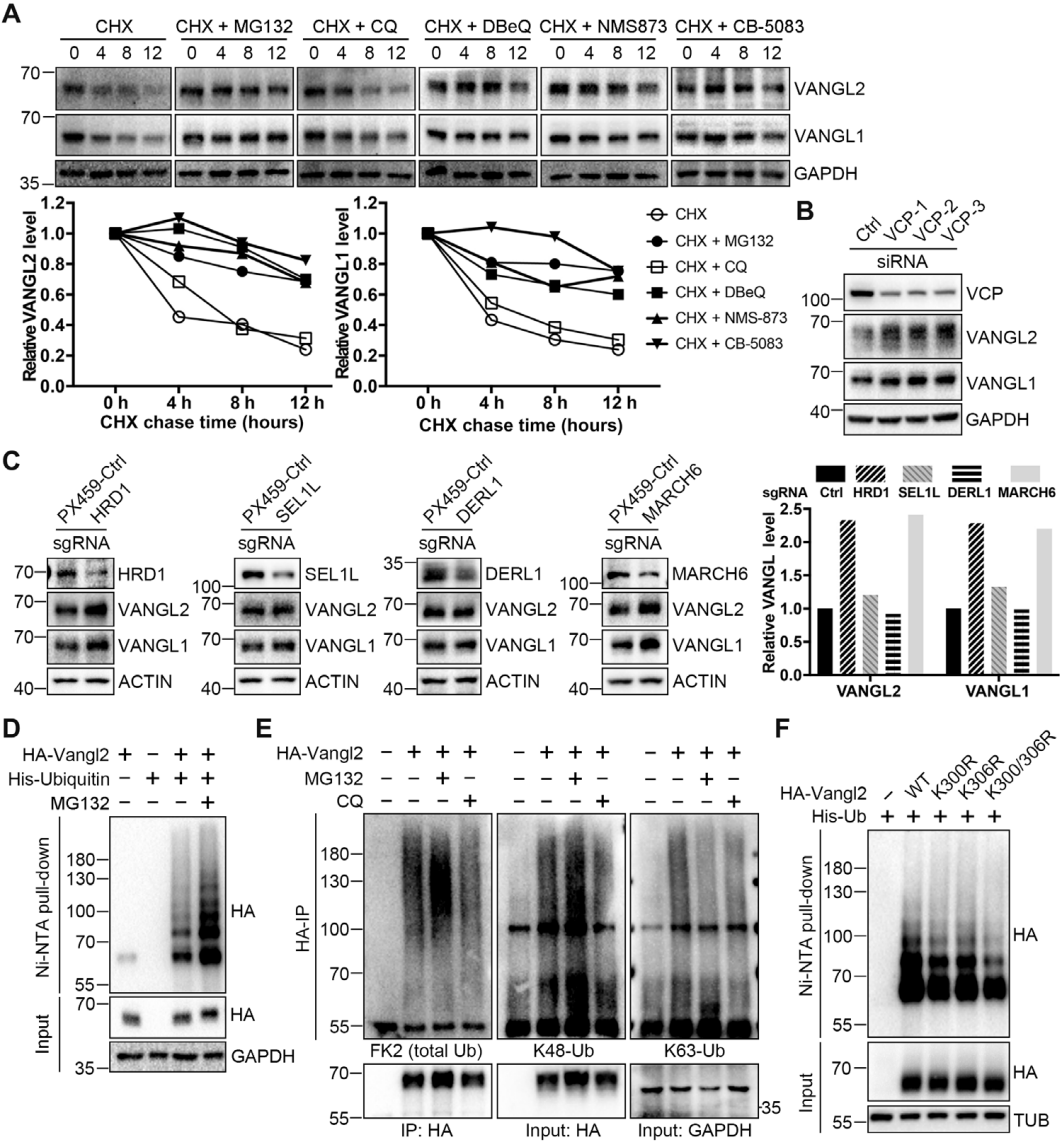
ERAD and ubiquitination of Vangl. (**A**) Proteasome and p97/VCP inhibitors inhibited the degradation of endogenous Vangl proteins. HEK293T cells were treated with CHX (100 μg/ml) alone or with proteasome inhibitor MG132 (10 μM), lysosome inhibitor CQ (25 μM) or p97/VCP inhibitor DBeQ (10 μM), NMS-873 (2 μM), or CB-5083 (5 μM) for the indicated time. Endogenous Vangl1 and Vangl2 were detected, and the relative Vangl protein levels were quantified below. (**B**) Knockdown of VCP by small interfering RNA (siRNA) increased Vangl protein levels in HEK293T cells. (**C**) Single guide RNA (sgRNA)–mediated knockdown of ERAD component HRD1 or MARCH6, but not SEL1L or DERL1, increased Vangl protein levels in HEK293T cells. The Vangl protein levels were quantified in the right panel. (**D**) Vangl2 was poly-ubiquitinated. Treatment of proteasome inhibitor MG132 (10 μM) for 4 hours significantly increased Vangl2 ubiquitination. (**E**) Vangl2 underwent K48-linked poly-ubiquitination. HEK293T cells were treated with MG132 (10 μM) or CQ (25 μM) for 4 hours, and ubiquitination was examined by total ubiquitin FK2, K48 linkage–, or K63 linkage–specific antibodies. The bands at 55 and 100 kDa were immunoglobulin G and nonspecific bands, respectively. Ub, ubiquitin. (**F**) K300 and K306 contribute to Vangl2 ubiquitination. The corresponding Vangl2 lysines (K) were mutated to arginines (R), which reduced the level of Vangl2 ubiquitination. GAPDH, glyceraldehyde 3-phosphate dehydrogenase.

Vangl proteins are synthesized in the ER and transported to the cell surface via the ER-to-Golgi secretory pathway and are then shuttled between the cell surface and endocytic vesicles (*50*–*52*). To examine the homeostatic regulation of Vangl proteins, we first treated human embryonic kidney (HEK) 293T cells with proteasome or lysosome inhibitors. We found that proteasome inhibitor MG132, rather than lysosome inhibitor chloroquine (CQ), was able to markedly increase endogenous Vangl protein levels (fig. S1A). We then analyzed protein stability by cycloheximide (CHX) chase assay. We observed substantial degradation of endogenous Vangl over a 12-hour period, which was largely rescued by MG132, whereas the lysosome inhibitor only delayed the degradation (Fig. 1A). This suggests that large amounts of endogenous Vangl proteins are normally degraded through the proteasomal pathway. Although membrane proteins are usually degraded via the lysosomal pathway, misfolded or unassembled membrane proteins can undergo proteasomal degradation via ERAD (*38*). The p97/VCP is a type II AAA+ adenosine triphosphatase essential for the extraction of substrates from the ER to the cytosol, which is a critical step in ERAD (*53*, *54*). We found that p97/VCP inhibitor (DBeQ, NMS-873, or CB-5083) attenuated the degradation of Vangl proteins (Fig. 1A). Knockdown of p97/VCP also significantly increased Vangl protein levels without affecting their mRNA levels (Fig. 1B and fig. S1B). Besides p97/VCP, recognition and extraction of ERAD substrates also require ERAD-specific components in the ER membrane, such as HRD1, SEL1L, DERL1, and DOA10/ MARCH6 (*36*–*38*). We found that knockdown of HRD1 or MARCH6 markedly increased Vangl protein levels, but knockdown of SEL1L or DERL1 had little or no effect (Fig. 1C). These results demonstrate that endogenous Vangl are mainly degraded in an ERAD-dependent manner.

As proteasomal degradation or ERAD is often preceded by ubiquitination, we next examined the ubiquitination status of Vangl2 under denaturing conditions. We found that Vangl2 was poly-ubiquitinated, and treatment of MG132 substantially increased ubiquitination (Fig. 1D). This was further confirmed by FK2, an ubiquitin-specific antibody, in both HEK293T and Chinese hamster ovary (CHO) cells (Fig. 1E and fig. S1C). Among the different types of ubiquitination, K48-linked poly-ubiquitination is well recognized as a targeting signal for the proteasomal degradation of substrates. We found that Vangl2 was mainly modified by K48-linked poly-ubiquitination chains (Fig. 1E and fig. S1C). Similarly, Vangl1 was also ubiquitinated mainly through K48 linkage (fig. S1, D and E). To determine the site(s) of ubiquitination in Vangl2, we first examined the ubiquitination status of truncated Vangl2, which revealed a critical region located at its C-terminal cytosolic tail between amino acids 254 and 521 (fig. S1, F and H). Sequence analyses and a proteomic database search suggested that the two clusters of highly conserved lysines, K300/K306 and K507/K510, were potential ubiquitination sites (fig. S1G). Mutagenesis studies of these sites (by generating lysine to arginine mutations) identified K300 and K306 as two important residues for Vangl2 ubiquitination, although additional sites could still contribute (Fig. 1F and fig. S1I). These findings imply the involvement of an ubiquitin-mediated mechanism in the regulation of Vangl proteins.

### KBTBD7 is a CUL3 substrate recognition protein for Vangl

**Fig. 2:**
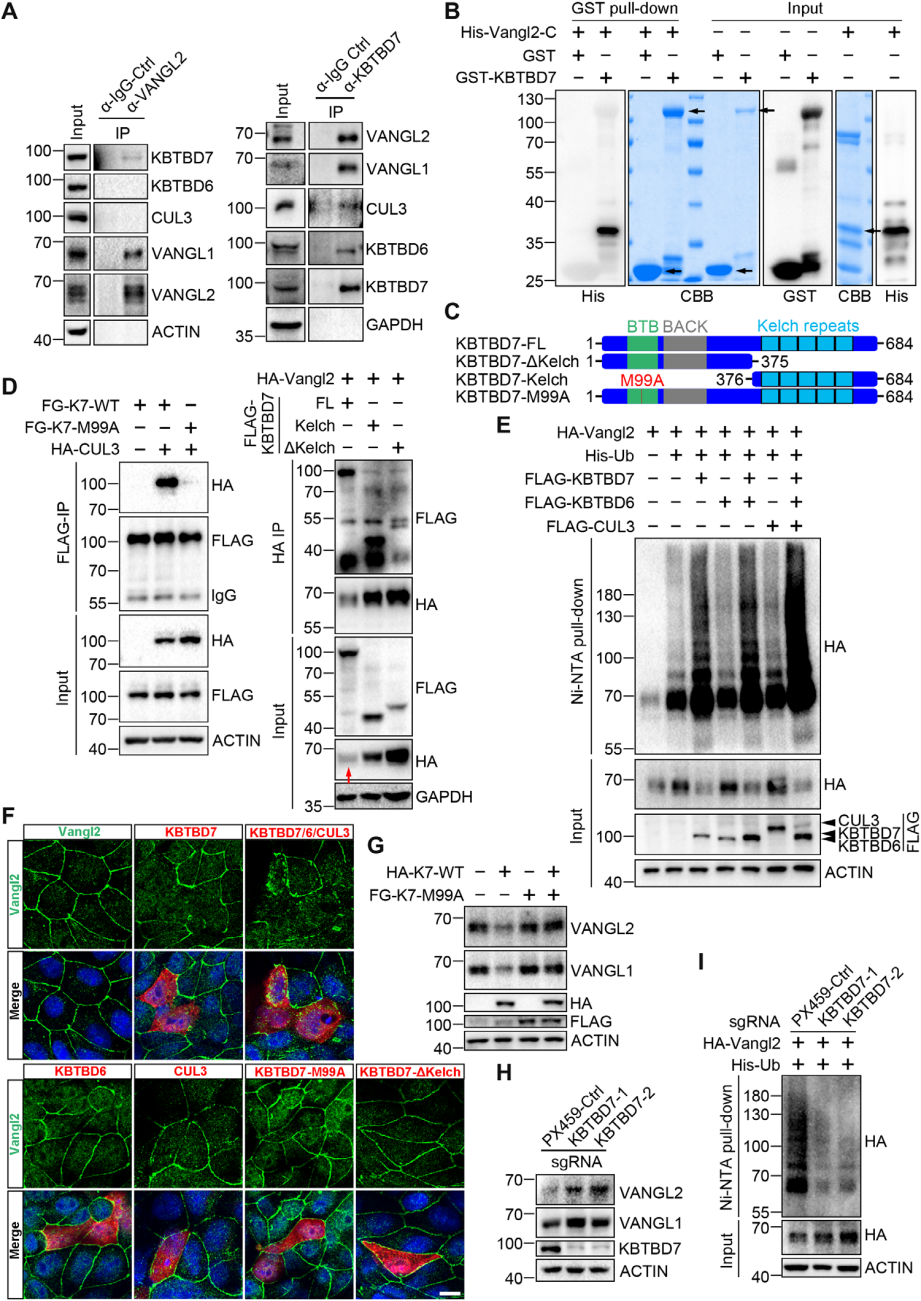
CUL3-KBTBD7 acts as an E3 ubiquitin ligase for Vangl. (**A**) Endogenous interaction between Vangl and KBTBD7 in HEK293T cells. Vangl2 binds to KBTBD7 and Vangl1 but not KBTBD6 or CUL3 (left). KBTBD7 binds to Vangl1, Vangl2, CUL3, and KBTBD6 (right). IgG, immunoglobulin G. (**B**) KBTBD7 directly binds to Vangl2. Glutathione *S*-transferase (GST)–fused KBTBD7 and His-fused C-terminal Vangl2 (amino acids 254 to 521) recombinant proteins were coincubated and subjected to GST pull-down. CBB, Coomassie brilliant blue staining. (**C**) Schematic domain structures of various KBTBD7. M99A, methionine to alanine mutation within the BTB domain. (**D**) Interaction between various Flag-tagged KBTBD7 (FG-K7) and HA-tagged CUL3 or Vangl2 in HEK293T cells. KBTBD7-M99A mutation abolished its binding to CUL3 (left). Kelch repeats of KBTBD7 were required for binding to Vangl2 (right). Note that overexpressed HA-Vangl2 was largely degraded by full-length KBTBD7 (red arrow in input). (**E**) KBTBD7 alone or with KBTBD6 and CUL3, but not KBTBD6 or CUL3 alone, promoted Vangl2 ubiquitination in HEK293T cells. (**F**) KBTBD7 abolished the plasma membrane localization of Vangl2. KBTBD6, CUL3, or mutant KBTBD7 was incapable of degrading Vangl2. Endogenous Vangl2 (green) in Madin-Darby canine kidney (MDCK) cells was examined by immunofluorescent staining (IF). The cells expressing KBTBD6, KBTBD7, or CUL3 were marked by FLAG IF (red). Nuclei were stained with 4′,6-diamidino-2-phenylindole (DAPI) (blue). Scale bar, 10 μm. (**G**) KBTBD7-M99A mutant was unable to degrade Vangl proteins but showed a dominant negative effect on wild-type (WT) KBTBD7 (K7). (**H**) Loss of KBTBD7 in HEK293T cells increased endogenous Vangl1 and Vangl2 protein levels. *KBTBD7* was knocked out in HEK293T cells by CRISPR-Cas9–mediated genome editing, and two single *KBTBD7* null clones were analyzed. The PX459 empty vector served as a control. (**I**) Loss of KBTBD7 decreased Vangl2 ubiquitination.

To identify the key molecules that directly regulate Vangl ubiquitination and stability, we performed affinity-based mass spectrometry (MS). FLAG-tagged Vangl2 was first transiently transfected into HEK293T cells. The Vangl2-containing protein complex was then pulled down by the anti-FLAG antibody and subjected to MS analysis, which found a number of ubiquitination-related proteins (fig. S2A). We then conducted coimmunoprecipitation (co-IP) to examine the interaction of each candidate with Vangl2. The co-IP results showed that KBTBD7 could strongly bind and degrade exogenously expressed Vangl2 (fig. S2, B and C). Both KBTBD7 and its close homolog KBTBD6 are substrate recognition proteins of CUL3 (*55*, *56*) and have been shown to form a CUL3-KBTBD6/7 complex (*56*). Overexpressed KBTBD6 and CUL3 could also bind Vangl2 but to a lesser extent (fig. S2B). KBTBD6 moderately reduced the level of Vangl2 but CUL3 had no effect (fig. S2B). We next examined endogenous interactions between Vangl and CUL3, KBTBD6, and KBTBD7 in HEK293T cells, which showed an interaction only between Vangl and KBTBD7, although KBTBD6 and KBTBD7 did bind to CUL3 and to each other (Fig. 2A and fig. S2D). This suggests that KBTBD7 is the main substrate recognition protein for Vangl. The in vitro pull-down of Vangl2 by KBTBD7 (but not KBTBD6) confirmed their direct interaction (Fig. 2B and fig. S2E). The failure of detection of endogenous and direct KBTBD6-Vangl interaction may reflect a very weak binding affinity between them, which was only manifested by overexpression (fig. S2B). To determine the binding domains of KBTBD7 and Vangl2, we generated a series of KBTBD7 and Vangl2 mutant constructs and examined their interactions (Fig. 2C and fig. S1F). Consistent with previous findings (*56*), we found a point mutation (M99A) in the BTB domain of KBTBD7 abolished its binding to CUL3 (Fig. 2D, left), indicating that the BTB domain mediates the interaction between KBTBD7 and CUL3. The KBTBD7-Kelch domain showed strong binding to Vangl2, whereas KBTBD7-∆Kelch mutant showed significantly weakened binding (Fig. 2D, right), indicating that Kelch repeats of KBTBD7 are responsible for the Vangl interaction. The binding between KBTBD7-M99A and Vangl2 was not affected (fig. S2F). Domain mapping of Vangl2 showed its C-terminal cytoplasmic tail (amino acids 254 to 521) was involved in its binding to KBTBD7 (fig. S2G). These results suggest that KBTBD7 is the key substrate recognition protein of CUL3, which may target Vangl for ubiquitination and degradation.

### CUL3-KBTBD7 ubiquitinates and degrades Vangl proteins

**Fig. 3:**
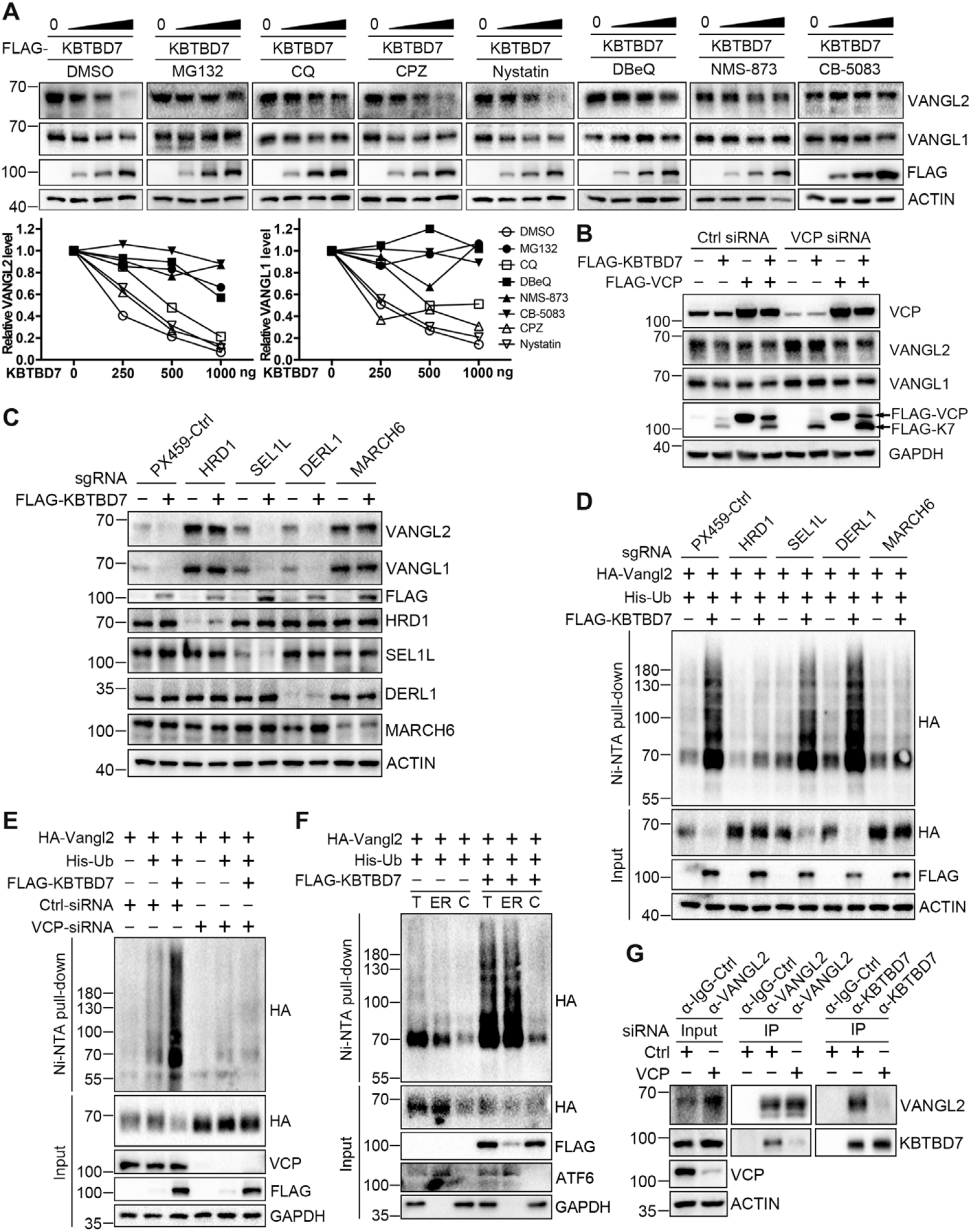
KBTBD7-mediated Vangl ubiquitination and degradation require ERAD machinery. (**A**) Inhibition of proteasome or p97/VCP attenuated the KBTBD7-induced Vangl degradation. HEK293T cells were transfected with KBTBD7 in an increasing dose (0, 250, 500, and 1000 ng) and treated with dimethyl sulfoxide (DMSO), MG132 (10 μM), CQ (25 μM), clathrin inhibitor CPZ (30 μM), caveolin inhibitor Nystatin (25 μg/ml) or p97/VCP inhibitor DBeQ (10 μM), NMS-873 (2 μM), or CB-5083 (5 μM) for 6 hours (4 hours for DBeQ). The relative Vangl protein levels were quantified below. (**B**) VCP is required for KBTBD7-induced Vangl degradation. Upon siRNA knockdown of VCP in HEK293T cells, KBTBD7 (K7) failed to induce Vangl degradation, which was restored by reconstitution of VCP. (**C**) HRD1 and MARCH6 are required for KBTBD7-induced Vangl degradation. sgRNA-mediated silencing of HRD1 or MARCH6, but not SEL1L or DERL1, protected Vangl from KBTBD7-induced degradation. (**D**) HRD1 and MARCH6 are required for KBTBD7-induced Vangl2 ubiquitination. sgRNA-mediated silencing of HRD1 or MARCH6, but not SEL1L or DERL1, blocked KBTBD7-induced Vangl2 ubiquitination in HEK293T cells. (**E**) VCP is required for KBTBD7-induced Vangl2 ubiquitination. KBTBD7 was unable to induce Vangl2 ubiquitination upon VCP knockdown by siRNA. (**F**) KBTBD7-induced Vangl2 ubiquitination mainly occurred in the ER but not in the cytosol. Total lysates, ER, and cytosolic fractions of HEK293T cells were isolated and subjected to ubiquitination assay. Activating transcription factor 6 (ATF6) served as an ER marker. T, total cell lysate; ER, ER fraction; C, cytosolic fraction. (**G**) The endogenous Vangl2-KBTBD7 interaction depends on VCP. Upon siRNA knockdown of VCP in HEK293T cells, the binding between Vangl2 and KBTBD7 was significantly reduced.

Given the interaction between KBTBD7 and Vangl, and that KBTBD7 significantly reduced the level of exogenously expressed Vangl2 (red arrows in Fig. 2D and fig. S2, B and C), we reasoned that CUL3-KBTBD7 might function as an E3 ligase to ubiquitinate and degrade Vangl proteins. We found that KBTBD7 alone strongly increased Vangl2 ubiquitination, whereas KBTBD6 or CUL3 alone had no significant effects (Fig. 2E). Expressing KBTBD6, KBTBD7, and CUL3 together gave rise to the highest level of Vangl2 ubiquitination (Fig. 2E). Accordingly, KBTBD7 promoted the degradation of endogenous Vangl in a dose- and proteasome-dependent manner, whereas KBTBD6 or CUL3 alone showed no or very little effects on the degradation of Vangl proteins (Figs. 2G and 3A and fig. S3A). Immunofluorescent staining in Madin-Darby canine kidney (MDCK) cells also demonstrated that KBTBD7 had a significant effect on the degradation of endogenous Vangl2. Cells expressing KBTBD7 lost Vangl2 staining on the cell surface (Fig. 2F). Consistent with the CUL3-KBTBD7–Vangl2 interaction results (Fig. 2D), KBTBD7-M99A and KBTBD7-∆Kelch mutants were unable to ubiquitinate and degrade Vangl proteins (Fig. 2, F and G, and fig. S3, A and B). The KBTBD7-M99A mutant even exhibited a dominant negative effect on wild-type (WT) KBTBD7 (Fig. 2G). These data indicate that CUL3 interaction and the Kelch domain are both required for KBTBD7’s function in promoting Vangl ubiquitination and degradation.

To further investigate the regulation of Vangl stability by KBTBD7, we generated *KBTBD7* knockout HEK293T cells by CRISPR-Cas9–mediated genome editing. Knockout of *KBTBD7* resulted in decreased Vangl2 ubiquitination and concomitantly increased Vangl abundance (Fig. 2, H and I). Reconstitution of KBTBD7, but not KBTBD7-M99A or KBTBD7-∆Kelch mutant, reduced Vangl2 expression in *KBTBD7* knockout cells (fig. S3C). The protein stability assay also showed that loss of KBTBD7 largely prevented the degradation of Vangl proteins (fig. S3D). Overall, our results showed that CUL3-KBTBD7 functions as a key E3 ligase for Vangl ubiquitination and degradation.

### KBTBD7-mediated Vangl ubiquitination and degradation require ERAD machinery

To understand how KBTBD7 degrades Vangl, we attempted to block KBTBD7-induced Vangl degradation by inhibiting different degradation routes. Lysosome inhibitor (CQ) slightly delayed the degradation, whereas clathrin or caveolin-mediated endocytosis inhibitors [chlorpromazine (CPZ) or Nystatin] had no effects. However, proteasome inhibitor MG132 largely prevented KBTBD7-induced Vangl degradation (Fig. 3A), indicating that KBTBD7 is involved in the degradation of Vangl via the ubiquitin-proteasome system, which is analogous to the constitutive degradation of Vangl in normal cells (Fig. 1A). Similarly, use of p97/VCP inhibitors (DBeQ, NMS-873, or CB-5083) (Fig. 3A) or knockdown of p97/VCP (Fig. 3B) also inhibited KBTBD7-induced Vangl degradation. Moreover, we found that the KBTBD7-induced Vangl ubiquitination and degradation also required the classical ERAD components HRD1 and MARCH6 (Fig. 3, C and D). Together, our data indicate that KBTBD7 ubiquitinates and degrades Vangl mainly through the ERAD pathway.

It is well known that p97/VCP can be recruited to the cytosolic surface of the ER through ER-resident cofactors, where it participates in ERAD with a collection of adaptors, including Ufd1-Npl4 core complex and many UBA-UBX proteins that bind directly to ubiquitin-modified substrates. Functional inhibition of p97/VCP blocked the extraction of ERAD substrates, leading to the accumulation of poly-ubiquitinated proteins (*53*, *54*, *57*). Unexpectedly, we found that KBTBD7 failed to induce Vangl2 ubiquitination when p97/VCP expression was silenced (Fig. 3E), indicating that p97/VCP is required for KBTBD7-mediated Vangl2 ubiquitination. Inspired by a recent study that reported a reubiquitination mechanism for p97/VCP-extracted membrane proteins (*58*), we suspected that KBTBD7 might also participate in reubiquitination of Vangl2 following p97/VCP-mediated extraction, in which the KBTBD7-induced Vangl2 ubiquitination is p97/VCP-dependent. To test this, we isolated ER membrane and cytosolic fractions of Vangl2 for ubiquitination assays. The results showed that KBTBD7-induced Vangl2 ubiquitination mainly occurs in the ER (Fig. 3F), which is unlikely to be a reubiquitination process. On the other hand, we found that p97/VCP knockdown but not p97/VCP inhibitor nearly abolished the endogenous interaction between Vangl2 and KBTBD7 (Fig. 3G and fig. S4A), suggesting that the physical presence but not the enzymatic activity of p97/VCP is required for KBTBD7 to access and ubiquitinate Vangl molecules in the ER.

### Vangl-p97/VCP direct interaction recruits KBTBD7 for Vangl ubiquitination

**Fig. 4:**
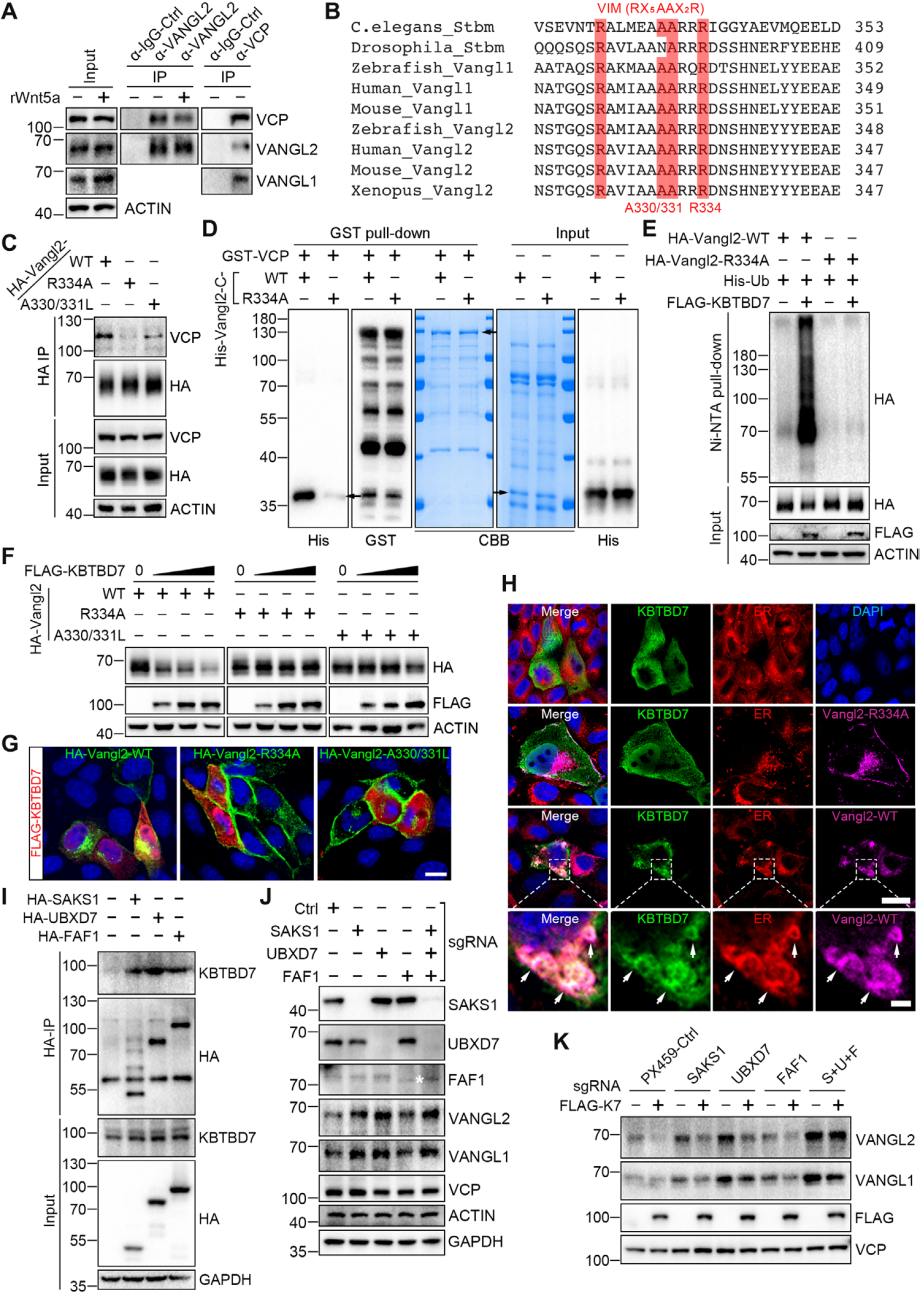
Vangl-p97/VCP direct interaction recruits KBTBD7 for Vangl ubiquitination. (**A**) Endogenous interaction between Vangl2 and VCP in HEK293T cells. rWnt5a, recombinant Wnt5a (200 ng/ml, 2 hours). (**B**) Sequence alignment identified a well-defined VCP-interacting motif (VIM) RX_5_AAX_2_R that is highly conserved across multiple species in both Vangl1 and Vangl2. (**C**) Mutations in Vangl2 VIM (R334A or A330/331L) nearly abrogated its interaction with p97/VCP. (**D**) VCP directly binds to Vangl2 via VIM. GST-fused VCP and His-fused C-terminal (amino acids 254 to 521) WT or VIM mutant (R334A) Vangl2 were purified, coincubated, and then subjected to GST pull-down. (**E**) KBTBD7 failed to induce the ubiquitination of VIM mutant (R334A) Vangl2. (**F**) VIM mutant Vangl2 (R334A or A330/331L) is resistant to the KBTBD7-induced degradation. HEK293T cells were transfected with an increasing dose (0, 250, 500, and 1000 ng) of KBTBD7. (**G**) KBTBD7 (red) failed to abolish the membrane localization of VIM mutant Vangl2 (R334A or A330/331L, green) in MDCK cells. Nuclei were stained with DAPI (blue). Scale bar, 10 μm. (**H**) KBTBD7 was enriched in the ER by WT Vangl2. KBTBD7 (green) was expressed with or without Vangl2 (WT or VIM mutant R334A, purple) in MDCK cells. The expression of WT but not VIM mutant Vangl2 led to an enrichment of KBTBD7 in the ER (arrows in the enlarged panel). ER was stained by ER dye (red). Scale bar, 10 μm in the top panels and 2 μm in the enlarged panel. (**I**) p97/VCP adaptor UBA-UBX proteins (SAKS1, UBXD7, or FAF1) bind to KBTBD7. (**J**) Knockdown of SAKS1 or UBXD7 by sgRNA significantly increased endogenous Vangl protein levels. (**K**) sgRNA-mediated knockdown of p97/VCP adaptors (SAKS1, UBXD7, and FAF1) compromised the KBTBD7-induced Vangl degradation.

The in cellulo requirement of p97/VCP for KBTBD7 to access Vangl2 (Fig. 3G) is unexpected, because KBTBD7 and Vangl2 can bind to each other in vitro independent of p97/VCP (Fig. 2B). To understand the underlying mechanism, we first examined the interaction between Vangl2 and p97/VCP by endogenous co-IP, which showed that they could also bind to each other (Fig. 4A). Intriguingly, Vangl proteins contain a well-defined VCP-interacting motif (VIM), RX_5_AAX_2_R, which is highly conserved from *Caenorhabditis elegans* to mammals (Fig. 4B) (*59*). WT Vangl2 can robustly interact with p97/VCP, whereas mutation of VIM either at the flanking arginine residue (R334A) or at the central alanine residues (A330/331L) nearly abrogated this interaction (Fig. 4C and fig. S4B). The in vitro pull-down of purified Vangl2 and p97/VCP proteins further confirmed their direct interaction via VIM (Fig. 4D). Unexpectedly, although mutation of VIM had no impact on Vangl2-KBTBD7 in vitro binding affinity (fig. S4C), KBTBD7 failed to induce ubiquitination in VIM mutant Vangl2 (Fig. 4E). VIM mutant Vangl2 was also resistant to KBTBD7-induced degradation (Fig. 4, F and G). Compared to WT Vangl2, VIM mutant Vangl2 still displayed strong membrane localization in KBTBD7-expressing cells (Fig. 4G). These results indicate that direct binding of p97/VCP to Vangl is required for KBTBD7-induced Vangl ubiquitination and degradation.

In light of the above findings, we speculated that the Vangl-p97/VCP interaction might recruit KBTBD7 to make it more accessible to Vangl molecules and stabilize the KBTBD7-Vangl interaction in the ER. The cytosolic KBTBD7 was not clearly localized in the ER in both MDCK and HEK293T cells (Fig. 4H and fig. S4D). We then coexpressed Vangl2 and KBTBD7 to assess the potential effects of Vangl on the localization of KBTBD7. In the presence of KBTBD7, most of the WT Vangl2 was degraded; whereas most of the VIM mutant Vangl2 was localized on the cell surface (Fig. 4G), we thus focused mainly on Vangl2 signals that were still detectable in the ER (Fig. 4H and fig. S4D). Expression of WT Vangl2 led to the enrichment of KBTBD7 in the ER (arrows in Fig. 4H and fig. S4D). However, when Vangl2 VIM was disrupted (Fig. 4H) or when p97/VCP expression was silenced (fig. S4D), the enrichment of KBTBD7 in the ER was abolished. These observations support our hypothesis that Vangl-p97/VCP interaction mediates the recruitment of KBTBD7. Nevertheless, how KBTBD7 is recruited remained unclear. The p97/VCP adaptor UBA-UBX proteins contain a UBX domain that interacts with the N terminus of p97/VCP and a UBA domain that binds ubiquitinated substrates (*53*, *54*, *57*). UBA-UBX proteins were also found associated with a number of ubiquitin ligases, for example, a network proteomics approach revealed that SAKS1, UBXD7, and FAF1 could possibly interact with KBTBD7 (*60*). We therefore examined the binding of these adaptor proteins to KBTBD7. We found that these three UBA-UBX proteins could efficiently pull-down endogenous KBTBD7 (Fig. 4I), suggesting a potential mechanism by which p97/VCP recruits KBTBD7 to Vangl molecules via its UBA-UBX adaptor proteins. The association of UBA-UBX proteins with KBTBD7 is unlikely mediated by the self-ubiquitination of KBTBD7 as KBTBD7-M99A which is defective in binding CUL3 was still strongly bound to SAKS1, UBXD7, and FAF1 (fig. S4E). Further investigation of these adaptors showed that silencing SAKS1, UBXD7, and FAF1 significantly increased Vangl protein levels (Fig. 4J) and compromised the ability of KBTBD7 to ubiquitinate and degrade Vangl (Fig. 4K and fig. S4F). In conclusion, our findings demonstrate an important role of p97/VCP in recruiting KBTBD7 to regulate Vangl proteins, which suggests a new mechanism of action of p97/VCP in the process of ERAD through substrate-p97/VCP direct interaction and subsequent recruitment of a cytosolic E3 ligase.

### Wnt5a inhibits Vangl ubiquitination via phosphorylation

**Fig. 5:**
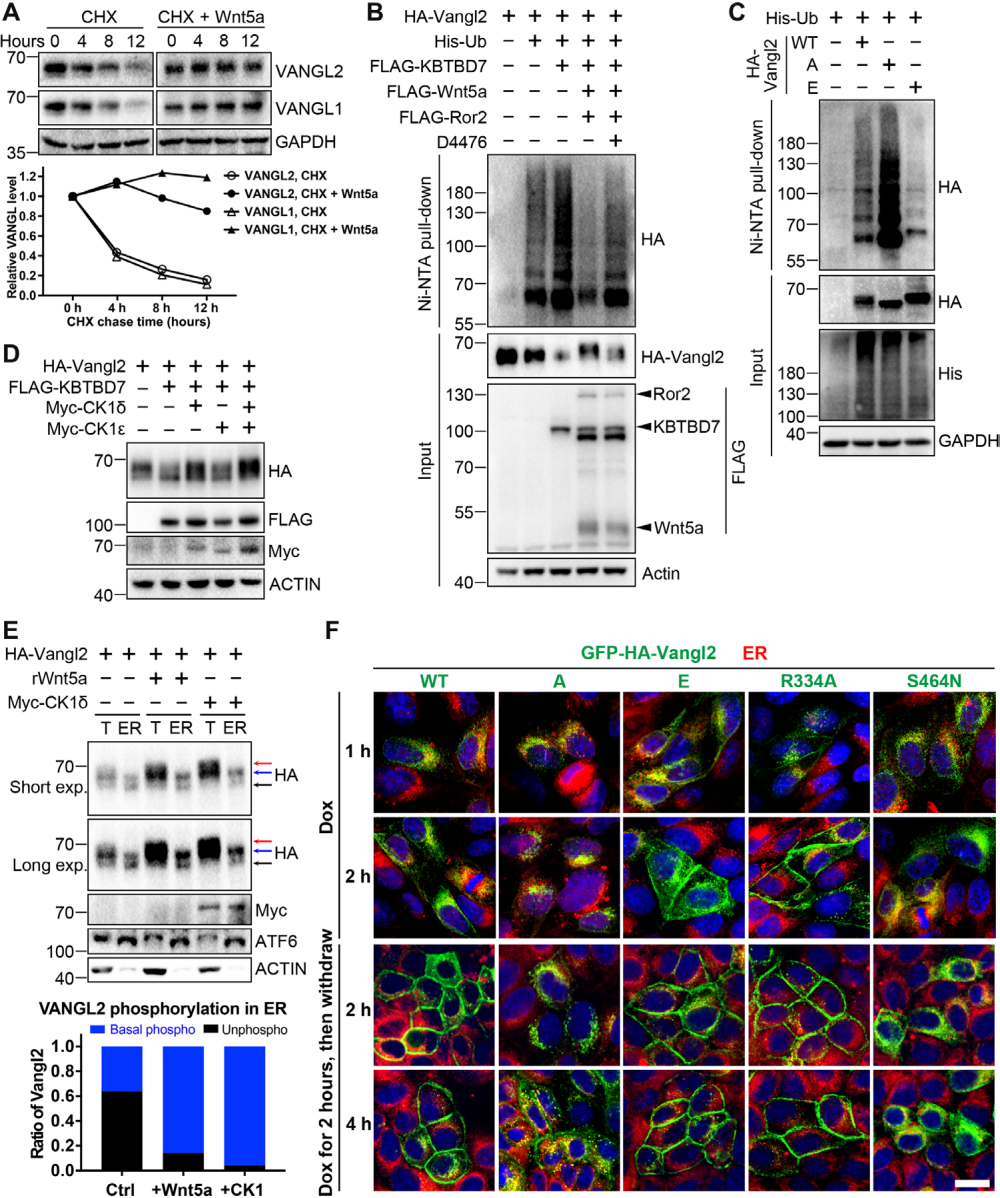
Wnt5a/CK1-induced Vangl phosphorylation inhibits ubiquitination and facilitates ER export of Vangl. (**A**) Wnt5a blocked the degradation of endogenous Vangl proteins. HEK293T cells were treated with CHX (100 μg/ml) alone or with rWnt5a (200 ng/ml) for the indicated time. The relative Vangl protein levels are quantified on the bottom panel. (**B**) Wnt5a inhibited KBTBD7-induced Vangl2 ubiquitination through CK1-mediated Vangl2 phosphorylation. CHO cells were treated with or without CK1 inhibitor D4476 (100 μM) for 6 hours. (**C**) Vangl2 phosphorylation inhibited its ubiquitination. HEK293T cells expressing WT, phospho-mutant (A), or phospho-mimetic (E) Vangl2 were treated with MG132 (10 μM) for 4 hours. Because phospho-mutant Vangl2 was known to be unstable, more plasmids were transfected to increase the input level. (**D**) CK1 protected Vangl2 from the KBTBD7-induced degradation. (**E**) Wnt5a and CK1 induced Vangl2 basal phosphorylation in the ER. CHO cells were treated with rWnt5a (200 ng/ml) for 2 hours or transfected with CK1δ. Vangl2 was examined in total cell lysates (T) and ER fractions (ER). ATF6 served as an ER marker. The lower black, middle blue, and upper red arrows point to the unphosphorylated, basal phosphorylated, and hyperphosphorylated Vangl2, respectively. The percentage of basal phosphorylated Vangl2 (blue) and unphosphorylated Vangl2 (black) in the ER fractions is quantified in the bottom panel. (**F**) Phosphorylation facilitated ER export of Vangl2. The transient expression of GFP-HA-tagged WT, phospho-mutant (A), phospho-mimetic (E), VIM mutant (R334A), or *Lp* mutant (S464N) Vangl2 (green) was induced by doxycycline (1 μg/ml) in MDCK stable cell lines for 1 or 2 hours (upper two rows). Doxycycline was removed after 2 hours of treatment, and the fate of Vangl2 (green) was traced for another 2 or 4 hours (lower two rows). ER, ER dye (red); nuclei, DAPI (blue). Scale bar, 10 μm.

We previously showed that Wnt5a can induce Vangl2 phosphorylation in two clusters of highly conserved serines and threonines through casein kinase 1 (CK1, mainly CK1ε and CK1δ) (*17*, *51*). Here, we found that treatment with Wnt5a proteins inhibited the degradation of endogenous Vangl proteins (Fig. 5A). We therefore further assessed the effects of Wnt5a and Vangl2 phosphorylation on ubiquitination. We found that Wnt5a, in combination with its receptor Ror2, strongly inhibited KBTBD7-induced ubiquitination of Vangl2, which could be partially reversed by the CK1 inhibitor D4476 (Fig. 5B). The phospho-mutant Vangl2 with all known phosphorylation sites mutated to alanines exhibited a higher level of ubiquitination, whereas a phospho-mimetic Vangl2 with glutamate substitutions exhibited a lower level of ubiquitination (Fig. 5C). These results indicate that the phosphorylation of Vangl2 can influence its ubiquitination. To unravel the underlying mechanism, we further investigated the interaction between Vangl2 and KBTBD7 with or without Wnt5a. We found that Wnt5a strongly decreased the interaction between Vangl2 and KBTBD7, which could be partially reversed by the CK1 inhibitor (fig. S5A), suggesting that Wnt5a may regulate Vangl2 ubiquitination through modulating the interaction between the substrate and E3 ligase in a phosphorylation-dependent manner. The interaction of endogenous Vangl2 and p97/VCP was also reduced with the Wnt5a treatment (Fig. 4A, middle). There are two possibilities: (i) Phosphorylation-mediated conformational change of Vangl2 directly prevents the binding of p97/VCP or KBTBD7, or (ii) phosphorylation-mediated subcellular localization changes in Vangl2 may result in less Vangl2 molecules available to p97/VCP or KBTBD7. To this end, we first performed in vitro pull-down assays using recombinant p97/VCP or KBTBD7 and cell lysates expressing WT, phospho-mutant, or phospho-mimetic Vangl2. We did not detect any significant differences in binding (fig. S5, B and C), which is consistent with the fact that Vangl2 binds to p97/VCP and KBTBD7 through VIM and its C-terminal tail, respectively, whereas the phosphorylations all occurred in the N-terminal region (Figs. 2B and 4D and fig. S2G). Therefore, it is unlikely that phosphorylation directly interferes with the binding of Vangl2 to p97/VCP or KBTBD7. On the other hand, Wnt5a treatment decreased cytoplasmic localization of Vangl2 but increased its plasma membrane localization (fig. S6A). Phospho-mimetic Vangl2 also showed preferential localization on the cell surface compared to phospho-mutant Vangl2 (fig. S6A). This could explain why Wnt5a reduces the interaction of Vangl with p97/VCP or KBTBD7, as there is a decreased amount of Vangl molecules available in the ER for binding to p97/VCP or KBTBD7. Overall, our data suggest that Wnt5a inhibits Vangl ubiquitination by inducing Vangl phosphorylation and promoting its membrane localization.

### Phosphorylation protects Vangl from ERAD

It was previously shown that the phospho-mutant Vangl2 was not stable in cultured cells and animal models (*51*). The *loop-tail* (*Lp*) mutant Vangl2 (carrying a well-known Vangl2 mutation such as D255E or S464N that causes the loop-tail phenotype in mice) was absent on the cell surface and largely degraded by proteasomes (*29*). To further understand how Vangl proteins are regulated, we first performed a Vangl2 internalization assay, which found that phospho-mutant Vangl2 was more easily endocytosed than WT Vangl2, with the phospho-mimetic Vangl2 having even lower endocytosis rate (fig. S5D). Although inhibiting lysosomal proteolysis stabilized phospho-mutant Vangl2, inhibiting proteasomes or p97/VCP further stabilized phospho-mutant Vangl2 to a greater extent (fig. S5E). Inhibiting proteasomes or p97/VCP also strongly stabilized *Lp* mutant Vangl2 (fig. S5E). These results suggest that misfolded or unmodified Vangl2 was preferentially recognized and degraded by the ERAD/proteasome pathway. Phospho-mutant Vangl2 was more readily found in the ER and proteasome (fig. S6B). Overexpression of KBTBD7 induced *Lp* and phospho-mutant Vangl2 degradation more effectively than in WT Vangl2 (fig. S5F), whereas loss of KBTBD7 significantly increased the protein levels (fig. S5G). In contrast, KBTBD7 did not induce phospho-mimetic Vangl2 degradation, and inhibition of p97/VCP or loss of KBTBD7 had no significant impact on its protein levels (fig. S5, E to G). Moreover, CK1-mediated phosphorylation of Vangl2 (CK1δ was more effective) also prevented KBTBD7-induced Vangl2 degradation (Fig. 5D). These data indicate that phosphorylation substantially protects Vangl from ERAD.

To investigate whether phosphorylation occurs in the ER, we isolated ER fractions by ultracentrifugation in the presence of Wnt5a or CK1, which showed a marked increase in the ratio of basal phosphorylated Vangl2 (middle blue arrow in Fig. 5E) to unphosphorylated Vangl2 (bottom black arrow in Fig. 5E). The phosphorylation shift of Vangl2 in the total cell lysates was more marked (top red arrow in Fig. 5E), likely reflecting further phosphorylation events induced by Wnt5a on the cell surface (*17*). Our data suggest that Wnt5a/CK1 are able to induce basal levels of Vangl2 phosphorylation in the ER, which may facilitate the export of Vangl2 from the ER to the plasma membrane, thereby escaping ERAD. To test this, we generated doxycycline-inducible MDCK stable cell lines to transiently express WT, phospho-mutant (A), phospho-mimetic (E), VIM mutant (R334A), and *Lp* mutant (S464N) Vangl2. Treatment with doxycycline for 2 hours induced low levels of Vangl2, which was comparable to the level of endogenous Vangl2 (fig. S6C). Consistent with previous findings (*17*, *51*), the phospho-mutant and *Lp* mutant Vangl2 exhibited dominant negative effects and reduced the level of endogenous Vangl2 (fig. S6C). We carefully examined the cellular localization of doxycycline-induced Vangl2 over time (Fig. 5F). The 1-hour doxycycline treatment induced Vangl2 expression, but it was mainly localized in the ER (only few phospho-mimetic or VIM mutant Vangl2 were presented on the cell surface) (Fig. 5F). After 2 hours of doxycycline treatment, a limited amount of WT Vangl2 appeared on the cell surface, with phospho-mimetic or VIM mutant Vangl2 showing very clear membrane localization, whereas phospho-mutant and *Lp* mutant Vangl2 were still retained in the ER (Fig. 5F). Next, we withdrew the doxycycline treatment to trace the fate of the different Vangl2. After 2 hours of withdrawal, many WT Vangl2 was presented on the plasma membrane, whereas phospho-mutant and *Lp* mutant Vangl2 were still localized in the ER (Fig. 5F). After 4 hours of withdrawal, some phospho-mutant Vangl2 could be observed on the cell surface, but *Lp* mutant Vangl2 was still trapped in the ER (Fig. 5F). The VIM mutant Vangl2 was very rapidly transported, which suggests a critical role of Vangl-p97/VCP interaction in this process. Inhibition of p97/VCP markedly increased the membrane localization of doxycycline-induced phospho-mutant Vangl2 (fig. S6D) and also largely restored membrane localization of endogenous Vangl2 abolished by KBTBD7 (fig. S6E). These observations suggest that the folding and assembly of Vangl proteins in the ER is a lengthy process and that the export of phosphorylated Vangl2 from the ER is very efficient, whereas the export of unphosphorylated Vangl2 is inefficient or delayed. Our data also suggest that blocking the extraction of Vangl by inhibiting p97/VCP may allow sufficient time for Vangl to be folded and assembled in the ER, which can eventually be exported from the ER to reach the cell surface. In contrast, the misfolded *Lp* mutant Vangl2 was not exported, and its ER retention could not be rescued by p97/VCP inhibitors (fig. S6D). Together, p97/VCP-mediated ERAD pathway appears to be a critical quality control mechanism that efficiently degrades misfolded or unfolded Vangl proteins to ensure only correctly folded and assembled molecules can reach the cell surface.

### Kbtbd7 degrades Vangl2 and causes CE defects in zebrafish

**Fig. 6:**
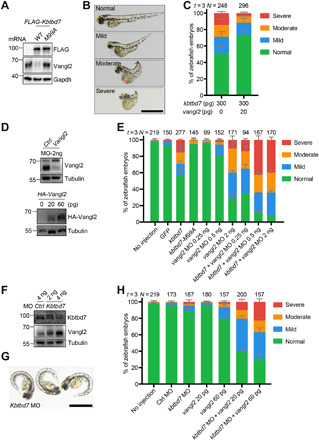
Kbtbd7 regulates Vangl2 in zebrafish convergent extension movement. (**A** to **C**) Kbtbd7 degraded Vangl2 and caused convergent extension (CE) defects in zebrafish. (A) Three hundred picograms of zebrafish *Kbtbd7* WT or M99A mutant mRNA was synthesized and microinjected into one-cell stage of embryos. Endogenous Vangl2 was examined. (B) Forty-eight hours after injection, zebrafish embryos were analyzed and classified into four groups (normal, mild, moderate, and severe) according to their CE phenotype. (C) Low level of Vangl2 (20 pg) partially rescued the CE defects caused by Kbtbd7 expression. The results are summarized from three independent experiments (*t* = 3). The total number of injected embryos (*N* =) for each group is labeled on the top. The error bars are the SD values. Scale bar, 1 mm. (**D**) The effects of Vangl2 MOs and *HA-Vangl2* mRNA in zebrafish embryos were examined by Vangl2 and HA immunoblotting, respectively. (**E**) Knockdown of *Vangl2* showed synergistic effects with *Kbtbd7* expression in causing CE defects. *Vangl2* MO (0.25, 0.5, or 2 ng) was injected alone or with *Kbtbd7* mRNA (300 pg). (**F**) Two or 4 ng of *Kbtbd7* MO was injected into one-cell stage of embryos. *Kbtbd7* knockdown increased endogenous Vangl2 protein levels in a dose-dependent manner. (**G**) Typical morphological defects of *Kbtbd7* MO–injected embryos, exhibiting ventral body curvature. Scale bar, 1 mm. (**H**) *Vangl2* expression exhibited synergistic effects with *Kbtbd7* MO in causing CE defects. Twenty or 60 pg of *Vangl2* mRNA was injected alone or with 4 ng of *Kbtbd7* MO. The results are summarized from three independent experiments (*t* = 3). The total number of injected embryos (*N* =) for each experimental setting is labeled on the top. The error bars are the SD values.

To test the in vivo functional significance of CUL3-KBTBD7 E3 ligase, we exploited the zebrafish model, which is a proven powerful and facile model system for the study of Wnt/PCP signaling pathway. The zebrafish CE phenotype, characterized by a shortened A-P axis, represents a rapid and sensitive functional readout for the Wnt/PCP signaling pathway (*5*). Either overexpression or loss of function of *Vangl2* can cause CE defects in zebrafish (*25*, *26*). Because CUL3 has essential functions in various fundamental cellular events and involves ~70 different substrate recognition proteins, manipulation of CUL3 will likely cause profound defects. Thus, we tested the function of *Kbtbd7* in zebrafish, which is the only zebrafish ortholog of human *KBTBD6* and *KBTBD7*. Injection of WT but not *M99A* mutant *Kbtbd7* mRNA into the one-cell embryos caused significant degradation of endogenous Vangl2 proteins (Fig. 6A) and obvious CE defects (Fig. 6, B, C, and E), whereas coinjection of 20 pg of *Vangl2* mRNA partially rescued the CE defects (Fig. 6C). This low-dose *Vangl2* did not cause CE defects in WT zebrafish (Fig. 6H). *Vangl2* antisense morpholino oligonucleotides (MOs) have been shown to cause robust CE defects (*25*, *26*). We found that 2 ng of *Vangl2* MOs caused severe CE defects, whereas low-dose *Vangl2* MOs (0.25 and 0.5 ng) had no or limited effects (Fig. 6, D and E). When *Kbtbd7* mRNA and low-dose *Vangl2* MOs were coinjected, significantly more severe CE defects were induced (Fig. 6E), demonstrating a synergistic effect of MO-mediated translational inhibition and *Kbtbd7*-mediated degradation of Vangl2 proteins.

We used MO injections to further test the loss-of-function effects of *Kbtbd7* on zebrafish gastrulation. The knockdown of *Kbtbd7* increased the level of endogenous Vangl2 in a dose-dependent manner (Fig. 6F), but this only resulted in a relatively mild morphological defect (Fig. 6G). This indicates that *Kbtbd7* negatively regulates Vangl2 in vivo, although the increase of Vangl2 was not sufficient to induce a strong CE defect compared to high-dose *Vangl2* mRNA (*25*, *26*). Zebrafish exhibited a phenotype of ventral body curvature (Fig. 6G), suggesting that knockdown of *Kbtbd7* may also perturb the level of other substrates that cause this phenotype. To further increase the Vangl2 level, we coinjected *Kbtbd7* MO with the low-dose *Vangl2* mRNA, which alone did not cause any apparent phenotype but, together with *Kbtbd7* MO, led to obvious CE defects (Fig. 6, D and H). These in vivo data demonstrate that Kbtbd7 plays an important role in regulating Wnt/PCP signaling through the core protein Vangl2.

### KBTBD7 inhibits breast cancer growth and metastasis

**Fig. 7:**
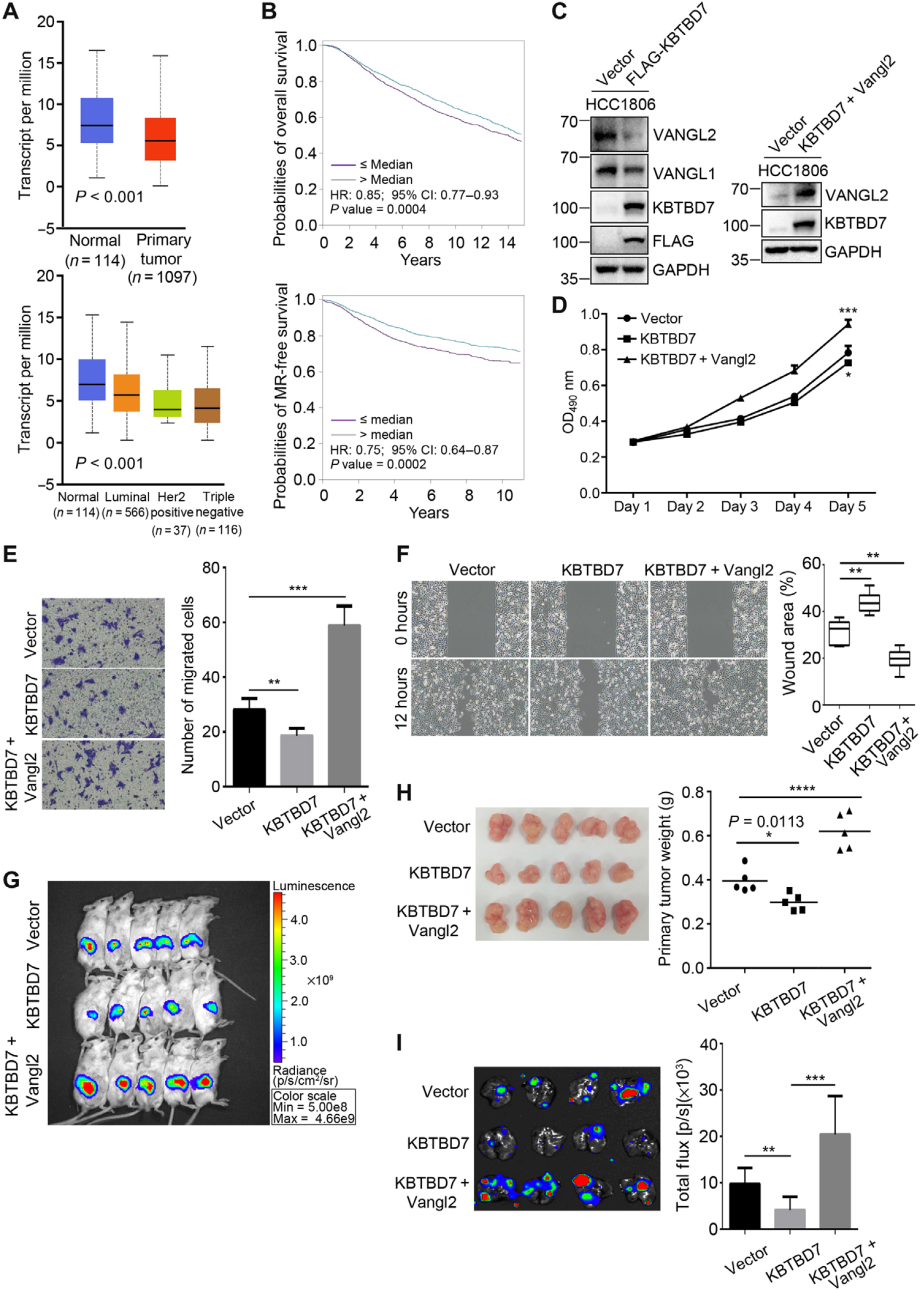
KBTBD7 inhibits growth and metastasis of mammary tumors. (**A**) TCGA data analysis of breast cancer. *KBTBD7* was down-regulated in breast carcinoma compared to normal breast tissues, Student’s *t* test, *P* < 0.001. (**B**) Kaplan-Meier survival analysis of 4295 patients for overall survival and 2637 patients for metastatic relapse–free (MR-free) survival (univariate Cox analysis, *P* = 0.0004 and 0.0002). Patients with lower *KBTBD7* expression level have decreased overall survival and MR-free survival. HR, hazard ratio. CI, confidence intervals. (**C**) Establishment of a HCC1806 breast cancer cell line stably expressing FLAG-tagged KBTBD7 (HCC1806-KBTBD7, left). Vangl2 was further stably expressed in HCC1806-KBTBD7 cells (right). (**D**) XTT assays of HCC1806 stable cancer cell lines. Expression of KBTBD7 slightly inhibited but further expression of Vangl2 promoted the proliferation of HCC1806 cells. (*n* = 3 repetitions; Student’s *t* test, *P < 0.05, ***P < 0.001). (**E** and **F**) Transwell migration (E) and wound healing (F) assays of HCC1806 stable cancer cell lines. KBTBD7 inhibited but Vangl2 promoted the migration of HCC1806 cancer cells (*n* = 3 repetitions; Student’s *t* test, **P < 0.01, ***P < 0.001). (**G** and **H**) KBTBD7 expression slightly inhibited but Vangl2 expression strongly promoted tumor growth in mouse xenograft models. (G) Ex vivo luciferase–based bioluminescence imaging of NOD SCID female mice 25 days after injection of HCC1806 stable cancer cells into the fat pads (*n* = 5 for each group). (H) Primary mammary tumors isolated from NOD SCID female mice 25 days after injection (left). Their body weights were measured (right) (*n* = 5 for each group; Student’s *t* test, *P = 0.0113, ****P < 0.0001). (**I**) Luciferase-based bioluminescence imaging (left) and quantification (right) of the lungs of NOD SCID female mice 8 weeks after injection of HCC1806 stable cancer cells into mammary fat pads (*n* = 4 for each group, Student’s *t* test, **P < 0.01, ***P < 0.001). KBTBD7 expression suppressed but Vangl2 expression strongly promoted lung metastasis of HCC1806 breast cancer cells.

Both Vangl1 and Vangl2 are found to be overexpressed in breast cancers and are associated with poor prognosis and have been implicated in tumor growth or cancer cell migration (*32*, *33*, *61*, *62*). Analysis of KBTBD7 in publicly available breast cancer TCGA (The Cancer Genome Atlas) datasets showed that *KBTBD7* was down-regulated in breast carcinomas compared to normal breast tissues (Fig. 7A). Further analysis in various subtypes of breast cancer also revealed a significant down-regulation of *KBTBD7* in luminal, HER2-positive, and triple-negative breast carcinomas (Fig. 7A). Survival analysis showed that patients with low *KBTBD7* expression level had decreased overall and metastatic relapse-free survival (Fig. 7B). These findings suggest that KBTBD7 may function as a tumor suppressor in breast cancers. To assess the functional importance of KBTBD7 in breast cancer, *KBTBD7* was stably expressed in HCC1806 cells, which is a triple-negative basal-like breast cancer cell line and known to express high levels of Vangl2 (*33*). Consistent with the findings in other cell types (e.g., HEK293T, CHO, and MDCK cells), expression of *KBTBD7* in HCC1806 cells led to the degradation of endogenous Vangl proteins, particularly Vangl2 (Fig. 7C). Next, we examined the effects of KBTBD7 on the proliferation and migration of breast cancer cells. The XTT assay showed that *KBTBD7* had a slight inhibitory effect on HCC1806 cell proliferation (Fig. 7D), whereas the transwell migration and wound healing assays showed that KBTBD7 had a significant inhibitory effect on breast cancer cell migration (Fig. 7, E and F). To further evaluate the tumor-suppressive effects of KBTBD7, we used a xenograft model to study tumor growth and metastasis. Control and KBTBD7-HCC1806 cells with a luciferase reporter were directly injected into the fat pad of nonobese diabetic/severe combined immunodeficient (NOD SCID) female mice. Mice injected with KBTBD7-HCC1806 cells showed a slight decrease in tumor growth (Fig. 7, G and H). Lung metastasis was detectable by bioluminescence imaging 8 weeks after tumor cell injection but was significantly blocked by *KBTBD7* expression (Fig. 7I), which is in line with KBTBD7 inhibiting breast cancer cell migration (Fig. 7, E and F). Furthermore, we expressed Vangl2 in KBTBD7-HCC1806 cells, so that KBTBD7 was not sufficient to degrade all exogenously expressed Vangl2 (Fig. 7C). These cells exhibited significantly increased cell proliferation and migration, and both tumor growth and lung metastasis were markedly enhanced (Fig. 7, D to I). Collectively, these results revealed that KBTBD7 plays an important suppressive role in breast cancer growth and metastasis through targeting Vangl proteins.

## DISCUSSION

**Fig. 8:**
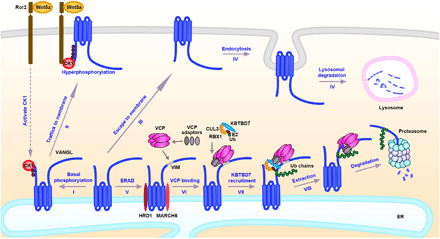
Model of ERAD-mediated regulation of Vangl. (I) Newly synthesized Vangl proteins are basally phosphorylated by CK1 in the ER; (II) the basal phosphorylated Vangl are transported to the cell surface where they undergo further phosphorylation and become stabilized; (III) some of unmodified Vangl escape ERAD and reach the cell surface; (IV) the unphosphorylated Vangl are not stable and are internalized and degraded via the lysosomal pathway; (V) most of the unmodified or unfolded Vangl proteins are degraded through the ERAD pathway. ERAD components HRD1 and MARCH6 are required for the degradation of Vangl and may initiate Vangl ubiquitination. (VI) VCP directly binds to Vangl at a highly conserved VIM; (VII) VCP recruits cytosolic E3 ligase KBTBD7 via its UBA-UBX adaptors (SAKS1, UBXD7, and FAF1), resulting in enhanced poly-ubiquitination of Vangl; and (VIII) extraction of highly ubiquitinated Vangl molecules for proteasomal degradation.

Genetic mutations or dysregulation of Wnt/PCP signaling has been implicated in a broad range of developmental defects and human diseases. Recent studies have indicated a strong link between aberrant Wnt/PCP activation and cancer development (*35*, *63*), which highlights the importance of the tight regulation in this signaling pathway. Accumulating evidence has revealed that the ubiquitination or deubiquitination of core proteins Dsh/Dvl, Pk, or Fmi is a critical regulatory mechanism in PCP (*41*–*45*). Ubiquitin-mediated regulation of Fz/Fzd has also been extensively studied but in the context of canonical Wnt/ß-catenin signaling (*64*–*67*). We showed here that KBTBD7, a CUL3 substrate recognition protein, functions as a negative regulator of Wnt/PCP signaling by regulating Vangl protein levels through ubiquitination. A key molecular feature of PCP is the asymmetric distribution of core proteins in planar polarized tissues, in which Fz/Dsh/Dgo and Vang/Pk protein complexes segregate to the opposite sides of the cell, and Fmi forms homodimers across neighboring cell membranes. Correct asymmetry is thought to be a result of feedback interactions between core proteins that amplify the initial symmetry-breaking signals (*3*, *68*). PCP is sensitive to the levels of its core proteins, and the establishment of PCP asymmetry largely depends on their delicate balance. Our studies identified CUL3-KBTBD7 as a key E3 ubiquitin ligase complex that can limit the amount of Vangl presented on the cell surface, thereby maintaining an appropriate level of Vangl relative to other core PCP proteins. We showed that ubiquitination of Vangl2 could be inhibited by Wnt5a/CK1-induced Vangl2 phosphorylation, which facilitated Vangl2 export from the ER to the plasma membrane (Fig. 8). This supports a critical role of Wnt signaling in regulating PCP. Furthermore, we demonstrated that KBTBD7 and Vangl2 synergistically caused CE defects in zebrafish embryos, which provides in vivo evidence that KBTBD7 is functionally important in the Wnt/PCP signaling pathway. In human breast cancer cells, prior studies have shown that Vangl1 and Vangl2 promote cancer cell proliferation or migration (*33*, *62*). Here, we found that expression of *KBTBD7* degraded Vangl and significantly suppressed mammary tumor metastasis in murine xenograft models, confirming its negative role in regulating Wnt/PCP signaling. Together, these findings reinforce the importance of maintaining appropriate levels of core PCP proteins in the Wnt/PCP signaling pathway.

Membrane proteins are usually degraded through two distinct routes: lysosomal degradation, which is the major pathway for the homeostatic regulation of transmembrane proteins, or ERAD, which is a quality control machinery that targets misfolded or unassembled proteins for proteasomal degradation (*36*, *38*). Unexpectedly, our results found that large amounts of WT Vangl molecules are normally degraded through the ERAD pathway, suggesting that the quality control of Vangl proteins occurs in the ER before they can reach the cell surface to function as key PCP signaling molecules. We unexpectedly identified a highly conserved VIM motif in both Vangl1 and Vangl2, which is responsible for Vangl-p97/VCP direct interaction. The p97/VCP plays a central role in mediating the degradation of target proteins by providing the driving force for the retrotranslocation or dislocation of ubiquitinated substrates from the ER to the cytosol (*53*, *54*). Most of proteins that directly interact with p97/VCP are either adaptors that link p97/VCP to a substrate or cofactors that facilitate substrate processing. Typically, p97/VCP acts through Ufd1-Npl4 or UBA-UBX adaptor proteins to bind ubiquitinated substrates (*53*, *54*, *57*). To the best of our knowledge, Vangl may be the first reported substrates that have a specific interacting motif for direct binding to p97/VCP. Besides the recognition and ubiquitination of misfolded/unfolded proteins by ER-resident ERAD components (e.g., HRD1 and MARCH6), cells may use the p97/VCP-substrate direct interaction to further anchor the ERAD substrates in the ER, enabling more efficient poly-ubiquitination by recruiting a more specific cytosolic E3 ligase. A possible scenario is that HRD1 and MARCH6 first initiate ubiquitination of Vangl, which is then further enhanced by KBTBD7 (Fig. 8). Similar collaboration between ER membrane–bound and cytosolic E3 ligases for ERAD has been recently reported (*69*, *70*). Although Vangl and KBTBD7 can physically bind to each other in vitro, the availability of KBTBD7 in the ER is obviously limited compared to p97/VCP, which is abundant in cells (~1% of total cellular proteins) and can be easily recruited to the ER through its ER-resident cofactors. Thus, recruitment of KBTBD7 by p97/VCP via its UBA-UBX adaptors may enable more efficient and stable KBTBD7-Vangl interaction in the ER, leading to enhanced ubiquitination and rapid degradation of Vangl proteins (Fig. 8). The proposed model is also supported by the fact that CUL3 alone was not sufficient to induce Vangl ubiquitination and degradation. The amount and recruitment of the CUL3 substrate recognition protein KBTBD7 are the limiting factors. p97/VCP adaptors UBXD7, FAF1, and SAKS1 were also found to be associated with CUL3 (*60*). Given that the CUL-RBX core module and E3 substrate recognition proteins may not always associate with each other (*71*), the p97/VCP adaptor–mediated binding of both CUL3 and KBTBD7 might stabilize the CUL3-KBTBD7 complex for Vangl ubiquitination. As p97/VCP adaptors are associated with a number of cytosolic ubiquitin ligases (*60*), it would be interesting to investigate whether there are any other substrates that p97/VCP can directly bind to and then recruit specific cytosolic E3 ligases for their ubiquitination. This is a potential mechanism whereby non–ER-resident E3 ligases are used for ERAD.

As Wnt5a/CK1 can induce Vangl2 phosphorylation in the ER and Vangl2 phosphorylation promotes its plasma membrane localization, phosphorylation may facilitate the folding or assembly of Vangl proteins and their exit from the ER. It was reported that Vangl could form homodimers or Vangl1/Vangl2 heterodimers (*29*). In vivo quantitative imaging studies in *drosophila* further implied a stoichiometry of six molecules of Vang to two molecules of Fmi and Fz in the core PCP protein complex (*72*). Thus, it is possible that Vangl proteins may need to be assembled as dimers or oligomers in the ER before being transported to the plasma membrane. It appears that ERAD functions as a gatekeeper to ensure that only properly folded and assembled Vangl molecules can efficiently exit the ER, while unfolded or unassembled proteins are subjected to ubiquitination and ERAD (Fig. 8). One possible mechanism is that correct folding and assembly may bury the VIM motif and thus reduce the binding of p97/VCP, whereas unfolded/unassembled Vangl would be recognized and bound by p97/VCP. Inhibition of p97/VCP or disruption of Vangl-p97/VCP interaction would allow these “unfolded/unassembled Vangl” to be folded and assembled. This explains why large amounts of WT Vangl molecules are degraded in an ERAD-dependent manner. The folding and assembly of WT membrane proteins are closely monitored by the ERAD machinery. If not folded/assembled in a timely fashion, they will also be targeted for degradation. Similar quality control mechanisms in the ER are involved in many other transmembrane proteins, e.g., the cystic fibrosis transmembrane regulator (CFTR), Wnt cargo receptor Evi (Wls/GPR177), and canonical Wnt signaling receptor low-density lipoprotein receptor-related protein 6 (LRP6) (*39*, *40*, *73*). It has been estimated that approximately 75% of the initially synthesized WT CFTR precursors are actually degraded via ERAD (*73*), and large amounts of newly synthesized WT LRP6 are also degraded in the ER (*39*). These observations indicate a critical role of ERAD in the quality control of complex transmembrane proteins, whereby lengthy folding and assembly or aberrant folding is targeted for ubiquitination and degradation. Although our findings identified ERAD as a critical quality control mechanism for PCP signaling molecules, further studies are needed to investigate whether Vangl are assembled as a complex and how Wnt signaling can affect this process.

The evolutionarily conserved gene *KBTBD7* encodes a BTB-Kelch family member that contains an N-terminal BTB domain, a C terminus containing five Kelch repeats, and an internal BACK domain. The BTB domain mediates the interaction with CUL3 and the Kelch domain mediates the interaction with substrates, which allow KBTBD7 to function as an adaptor of CUL3-RING ligase (CRL3) for recruiting target proteins for ubiquitination and degradation. For example, CUL3-KBTBD7 has been shown to destabilize neurofibromin 1 (NF1) in glioblastoma cells, making it a potential therapeutic target for glioblastoma (*55*). The close homolog of KBTBD7, KBTBD6 (sharing ~90% sequence identity), can form a heterodimeric complex with KBTBD7 to ubiquitinate TIAM1 (T-lymphoma and metastasis gene 1) and DRD2 (dopamine receptor D2) (*56*, *74*). However, in contrast to KBTBD7, the role of KBTBD6 in the regulation of Vangl is very limited and no endogenous interaction between KBTBD6 and Vangl has been detected, indicating that KBTBD7 is the major regulator of Vangl proteins. TIAM1 is a Rac1-specific GEF (guanine exchange factor), and Rac1 has been reported to mediate the effects of Vangl2 on the actin cytoskeleton (*75*, *76*). Nevertheless, KBTBD7-induced CE defects in zebrafish are unlikely to be mediated by the TIAM1-Rac1 signaling axis, because no CE phenotype was reported in *Rac1* knockdown or knockout zebrafish (*77*, *78*). However, *Vangl2* knockdown or null zebrafish exhibited severe CE defects (*25*, *26*), and KBTBD7-induced CE defect could be rescued by *Vangl2* expression, which supports Vangl2 as an important functional target of KBTBD7 in zebrafish gastrulation. Moreover, KBTBD7 was found to be involved in inflammation and cardiac dysfunction as a direct target of miR-21 (*79*), but the precise role of *KBTBD7* in mammals remains unclear.

Notably, KBTBD7 appears to have both tumor-promoting and tumor-suppressing functions. In glioblastoma cells, KBTBD7 destabilized NF1 tumor suppressor, suggesting that it has an oncogenic role in the pathogenesis of glioblastoma (*55*). Moreover, KBTBD7 was found to decrease pituitary tumor sensitivity to dopamine agonist treatment through DRD2 (*74*). However, our analysis of breast cancer datasets and our studies on breast cancer cells and xenograft models revealed that KBTBD7 had a suppressive role on breast cancer progression. Recently, *KBTBD7* was also identified as a protective gene in an eight-gene prognostic signature for early-stage non–small cell lung carcinoma (*80*). cBioPortal data analysis showed that *KBTBD7* is deleted or mutated in a considerable number of prostate, bladder, and uterine cancer patients, further implicating its tumorigenic relevance in other cancer types and suggesting a potential tumor-suppressing function. Therefore, the findings presented here could provide mechanistic rationale for the development of new therapeutic strategies for various cancers that show aberrations in the Wnt/PCP signaling pathway.

## MATERIALS AND METHODS

### Plasmids

The *Vangl2*, *Wnt5a*, *Ror2*, and *CK1* expression plasmids have been described previously (*17*, *51*). *KBTBD6*, *KBTBD7*, and *CUL3* cDNAs were reverse transcribed from HEK293T cells and cloned into pCMV-3Tag-1A (3xFLAG) plasmid at BamHI/EcoRI (for *KBTBD6* and *KBTBD7*) and SrfI/XhoI sites (for *CUL3*), respectively. *Vangl2* and *KBTBD7* truncated plasmids were generated on the basis of their respective full-length plasmids. All site-mutated plasmids were generated using a standard site-directed mutagenesis method. The His-Vangl2-C-WT construct was made by inserting the Vangl2 C-terminal intracellular domain (amino acids 254 to 521) into pET28a at EcoRI/XhoI sites. The glutathione S-transferase (GST)–KBTBD6 and GST-KBTBD7 constructs were generated by inserting *KBTBD6* and *KBTBD7* cDNA into the pGEX-6P-1 plasmid at EcoRI/XhoI sites. The pcDNA6-N-3xFLAG-VCP was a gift from B. Beutler (Addgene #123600). The GST-VCP construct was generated by inserting *VCP* cDNA into the pGEX-6P-1 plasmid at BamHI/SalI sites. The pcDNA5FRT/TO-SAKS1-Strep-HA (Addgene #113487), pcDNA5FRT/TO-UBXD7-Strep-HA (Addgene #113479), and pcDNA5F.RT/TO-FAF1-Strep-HA (Addgene #113486) were gifts from H. Meyer. To generate doxycycline-inducible GFP-HA-Vangl2 plasmids, different forms of HA-Vangl2 (WT, phospho-mutant, phospho-mimetic, R334A, and S464N) were inserted into pCW57-GFP-2A-MCS (Addgene #71783) at AgeI and BamHI (2A site was deleted). To generate HEK293T knockout or knockdown cells, two pairs of single guide RNA (sgRNA) oligos were used for *KBTBD7*, *HRD1*, *SEL1L*, *DERL1*, *MARCH6*, *SAKS1*, *UBXD7*, and *FAF1*. The sgRNA sequences are listed in Supplementary Materials and Methods. The annealed oligos were inserted into the PX459 plasmid at Bbs I site, which was a gift from F. Zhang (Addgene #48139). Zebrafish *Kbtbd7* cDNAs were synthesized and inserted into pCMV-3Tag-1A plasmid at EcoRV/XhoI sites. Lentiviral expression constructs of *Vangl2* and *KBTBD7* were made by inserting *HA-Vangl2* and *FLAG-KBTBD7* into the pCDH-CMV-MCS-EF-1α-copGFP-T2A-Puro plasmid (System Biosciences) at XbaI/EcoRI sites. The pLenti-EF1a-Luciferase-IRES-Blast-WPRE was a gift from J. Alcudia (Addgene #108542).

### Generation of stable cell lines

To generate *KBTBD7* knockout cells, HEK293T cells were transfected with PX459-KBTBD7-sgRNA plasmids for 48 hours, followed by selection with puromycin (2 μg/ml) until most cells from nontransfected control group had died. Single colonies were then picked up and characterized for KBTBD7 expression. To generate doxycycline-inducible stable MDCK cells that express different forms of GFP-HA-Vangl2, pCW57-GFP-HA-Vangl2 constructs were first packaged with psPAX2 packaging vector and pMD2.G envelope vector into HEK293T cells for 72 hours. MDCK cells were then infected with the above viral media in the presence of Polybrene (8 μg/ml) for 2 days. Puromycin (2 μg/ml) was used for 3 days to select transduced-positive cells. The established MDCK cell lines were treated with doxycycline (1 μg/ml) to induce Vangl2 expression. To generate HCC1806 cells that stably express *KBTBD7* and *KBTBD7* + *Vangl2* with constitutive luciferase expression, pLenti-EF1a-Luciferase-IRES-Blast-WPRE construct was first packaged with psPAX2 packaging vector and pMD2.G envelope vector into HEK293T cells for 72 hours. Media containing the above lentivirus were collected and centrifuged at 3500 rpm for 15 min to remove cell debris and then filtered through 0.45-μm filters (Millipore). HCC1806 cells were then infected with the above viral media in the presence of Polybrene (8 μg/ml) for 2 days. Blasticidin (2 μg/ml) was used for selection of transduced-positive cells for incubation for 3 days. Luciferase stably expressing HCC1806 cells were further infected with pCDH-CMV-MCS-EF-1α-copGFP-T2A-Puro empty vector, FLAG-KBTBD7, and/or HA-Vangl2 expressing lentivirus in the presence of Polybrene (8 μg/ml) for 2 days, followed by puromycin (2 μg/ml) selection for 3 days. These steps generated *KBTBD7* and *KBTBD7* + *Vangl2* HCC1806 stable cell lines with constitutive luciferase expression, which were maintained in media containing puromycin (0.5 μg/ml).

### Antibodies, inhibitors, antibiotics, siRNA, and recombinant proteins

The antibodies used in this study are listed in Supplementary Materials and Methods. The chemical inhibitors used in this study include protein synthesis inhibitor CHX (100 μg/ml; Sigma-Aldrich, #01810), proteasomal degradation inhibitor MG132 (10 μM; Abcam, ab141003), lysosomal degradation inhibitor CQ (25 μM; Sigma-Aldrich, C6628), p97/VCP inhibitor DBeQ (10 μM; Sigma-Aldrich, SML0031), NMS-873 (2 μM; Sigma-Aldrich, SML1128) or CB-5083 (5 μM; MedChem Express, HY-12861), clathrin inhibitor CPZ (30 μM; Sigma-Aldrich, C8138), caveolin inhibitor Nystatin (25 μg/ml; Sigma-Aldrich, N6261), CKI inhibitor D4476 (100 μM; Abcam, ab120220), and deubiquitinase inhibitor *N*-ethylmaleimide (NEM; 10 mM; Thermo Fisher Scientific, #23030). Other reagents used in this study include puromycin (2 μg/ml; Invivogen, ant-pr-1), blasticidin (2 μg/ml; Invivogen, ant-bl-05), VCP small interfering RNA (siRNA) (sc-37187, Santa Cruz Biotechnology), and recombinant Wnt5a proteins (R&D Systems, 645-WN).

### Ubiquitination assay

Vangl ubiquitination assay was performed under denaturing conditions (either 2% SDS or 6 M guanidine) to avoid co-IP of interacting proteins and to ensure the ubiquitin signal originated from only Vangl. To detect endogenous ubiquitination, cells were transfected with HA-Vangl2, lysed with radioimmunoprecipitation assay (RIPA) lysis buffer [50 mM tris-HCl (pH 7.4), 150 mM NaCl, 1 mM EDTA, 0.25% deoxycholic acid, and 1% NP-40] containing 2% SDS, 10 mM NEM, and protease and phosphatase inhibitors (Roche, cOmplete, EDTA-free Protease Inhibitor Cocktail, #11873580001 and Roche, PhosSTOP, #4906845001), and then boiled at 95°C for 10 min, followed by brief sonication until a clear solution was obtained. The lysates were centrifuged (16,000*g*, 4°C, 15 min), and supernatants were diluted 10 times with additional RIPA lysis buffer to reduce the SDS concentration to 0.2%. The supernatants were then incubated with anti-HA antibody at 4°C overnight. Protein A/G PLUS-Agarose beads (Santa Cruz Biotechnology, sc-2003) were prewashed with phosphate-buffered saline (PBS) twice and RIPA lysis buffer once and then added to the supernatants for further incubation at 4°C for 2 hours. Beads were then washed four times in RIPA lysis buffer containing 0.1% SDS. After the last wash, 1× Laemmli sample buffer (Bio-Rad, #1610747) containing 10% 2-mercaptoethanol was added to the beads and heated at 95°C for 10 min. Immunoprecipitated HA-Vangl2 and coimmunoprecipitated ubiquitin were separated by 10% SDS–polyacrylamide gel electrophoresis (PAGE) and subjected to immunoblotting. Blots were developed using SuperSignal West Femto Chemiluminescent Substrate (Thermo Fisher Scientific, PI34096) and digitally detected using ChemiDoc MP Imaging System (Bio-Rad) and analyzed with Image Lab software. For the His-ubiquitin pull-down assay, the following procedures were used. Cells were transfected with His-ubiquitin and the indicated plasmids for 48 hours and then lysed with Buffer A [6 M guanidine-HCl, 0.1 M Na_2_HPO_4_/NaH_2_PO_4_, and 10 mM imidazole (pH 8.0)] containing 10 mM NEM, protease and phosphatase inhibitors, followed by brief sonication until a clear solution was obtained. The lysates were centrifuged (16,000*g*, 4°C, 15 min), and supernatants were then incubated with nickel–nitrilotriacetic acid (Ni-NTA) beads (QIAGEN) for 3 hours at room temperature. Beads were then washed once in Buffer A, twice in Buffer B (a mixture of Buffer A:Buffer TI at a ratio of 1:3), and once in Buffer TI [25 mM tris-HCl and 20 mM imidazole (pH 6.8)]. After the last wash, 1× Laemmli sample buffer containing 10% 2-mercaptoethanol was added to the beads and heated at 95°C for 10 min. The ubiquitinated proteins were then analyzed by immunoblotting.

### GST and His-fusion protein purification and pull-down

For GST-fused protein purification, pGEX-6P-1 empty (GST), pGEX-6P-1-KBTBD6 (GST-KBTBD6), pGEX-6P-1-KBTBD7 (GST-KBTBD7), and pGEX-6P-1-VCP (GST-VCP) plasmids were transformed into *Escherichia coli* BL21 strain. A single colony was picked up and cultured in 5 ml of LB containing ampicillin (LA) (0.1 μg/μl) overnight. Then, 4 ml of cultured *E. coli* was inoculated into a large volume of LA (400 ml) until OD at 600 nm reached 0.6. Expressions of GST, GST-KBTBD6, GST-KBTBD7, and GST-VCP proteins were then induced with 200 μM isopropyl-β-d-thiogalactopyranoside (IPTG) for 16 to 20 hours at 16°C. Bacteria were harvested by centrifugation (4000*g*, 4°C, 15 min) and resuspended in 40 ml of bacteria lysis buffer [20 mM tris-HCl (pH 7.4), 150 mM NaCl, 10 mM EDTA, 1% Triton X-100, and 1 mM dithiothreitol (DTT) containing protease inhibitors]. Bacteria were sonicated for 30 min (on for 3 s and off for 7 s) at the 50% amplitude until completely lysed. Cell debris were removed by centrifugation (16,000*g*, 4°C, 20 min), and the supernatant was incubated with 1 ml of Pierce Glutathione Agarose beads (Thermo Fisher Scientific, #16100) with gentle rotation at 4°C overnight. Glutathione agarose beads were then loaded onto Poly-Prep Chromatography Columns (Bio-Rad, #7311550) for column binding. GST protein–bound glutathione agarose beads were washed with 50 ml of cold wash buffer [20 mM tris-HCl (pH 7.4), 150 mM NaCl, 10 mM EDTA, and 1 mM DTT] and eluted with 5 ml of elution buffer {PBS [137 mM NaCl, 10 mM phosphate, and 2.7 mM KCl (pH 7.4)] containing 50 mM glutathione}.

For His-fused protein purification, the His-Vangl2-C-WT (amino acids 254 to 521) and His-Vangl2-C-R334A (amino acids 254 to 521) plasmids were transformed into *E. coli*. BL21 strain and cultured in LB with kanamycin (50 μg/ml), following similar procedures. Buffer used for His-tagged protein purification was based on the NTA basal buffer [50 mM NaH_2_PO_4_, 500 mM NaCl, and 10% glycerol (pH 8.0)]. After IPTG induction and centrifugation, bacteria were resuspended in the lysis buffer (NTA10PCT, NTA basal buffer and 10 mM imidazole, 0.5% Triton X-100, and 1 mM DTT containing protease inhibitors). After sonication, the supernatant was incubated with 1 ml of Ni-NTA beads (QIAGEN) with gentle rotation at 4°C overnight. The Ni-NTA beads were then loaded onto Poly-Prep Chromatography Columns (Bio-Rad, 7311550) for column binding. His-fused protein-bound Ni-NTA beads were washed with 25 ml of cold wash buffer I (NTA20PCT, NTA basal buffer and 20 mM imidazole, 0.5% Triton X-100, and 1 mM DTT containing protease inhibitors) and 25 ml of cold wash buffer II (NTA20 and NTA basal buffer and 20 mM imidazole) and then eluted with 2 ml of elution buffer (NTA500 and NTA basal buffer and 500 mM imidazole).

For in vitro pull-down experiments, equal amounts of purified GST-fused proteins were incubated with 200 μl of fresh glutathione agarose beads with gentle rotation at 4°C for 2 hours. After incubation, equal amounts of purified His-fused proteins were added to GST-fused protein-bound glutathione agarose beads and further incubated overnight with gentle rotation. Glutathione agarose beads were washed five times with 1 ml of cold wash buffer and eluted with 1× Laemmli buffer containing 10% 2-mercaptoethanol. The pulled-down proteins were separated by 10% SDS-PAGE and subjected to Coomassie brilliant blue staining and immunoblotting.

### Immunofluorescence and confocal microscopy

For immunofluorescent staining, MDCK or HEK293T cells grown on slides were transfected with the indicated plasmids for 48 hours and treated with the indicated inhibitors (as needed) before fixation. For ER staining, ER dye (red) from the ER staining kit–Red Fluorescence | Cytopainter (Abcam, ab139482) was used at 1:1000 dilution, and the cultured cells were incubated for 30 min before fixation according to the manufacturer’s instructions. Cells were rinsed twice with cold PBS and then fixed with 4% paraformaldehyde/PBS at 4°C for 10 min, followed by permeabilization with 0.5% Triton X-100/PBS for 5 min and then blocked with 3% bovine serum albumin in 0.1% Triton X-100/PBS for 1 hour at room temperature. Cells were then incubated with the indicated primary antibodies at 4°C overnight. After rinsing three times for 5 min with PBS, secondary antibodies bound to Alexa Fluor 488, 568, or 647 (Thermo Fisher Scientific) were diluted at 1:1000 and incubated at room temperature for 1 hour. After rinsing three times with PBS, DAPI (4′,6-diamidino-2-phenylindole; 300 nM; Thermo Fisher Scientific, D1306) was added and further incubated at room temperature for 5 min. Slides were then mounted with ProLong Diamond Antifade Mountant (Thermo Fisher Scientific, P36965) and examined by Zeiss LSM780 confocal microscopy. Images of violet, green, red, and far-red signals were detected by excitation wavelengths of 405, 488, 568, and 647 nm, respectively, and analyzed by Zeiss Zen software. ImageJ software (National Institutes of Health, v1.52q) was used to quantify the membrane and cytosolic signals of Vangl2. Signal intensities of the entire cell and intracellular fluorescence were measured after calibration of background signals around the cell. The membrane signal intensities were calculated by subtracting the intracellular intensities from the total cell intensities.

### ER fraction enrichment

ER fractions were isolated using the Endoplasmic Reticulum Enrichment Kit (Novus Biologicals, NBP2-29482) according to the manufacturer’s instructions. The CHO cells in a 10-cm dish were transfected with the indicated plasmids for 48 hours and treated with rWnt5a (200 ng/ml) for 2 hours before cell harvesting. Cells were homogenized in 1 ml of 1× isosmotic homogenization buffer containing protease and phosphatase inhibitors with 20 strokes. Nuclei fraction and cell debris were removed by centrifugation (1000*g*, 4°C, 10 min), and supernatants were centrifuged (12,000*g*, 4°C, 15 min) again to remove mitochondria fraction and cell debris. Supernatants were further ultracentrifuged (100,000*g*, 4°C, 60 min), and the ER fraction pellets were dissolved in 1× Laemmli buffer containing 10% of 2-mercaptoethanol, then separated by 10% SDS-PAGE, and subjected to immunoblotting.

### Zebrafish assay

All zebrafish experiments in this study were conducted in compliance with the Guidelines from The Committee on Use of Laboratory Animals for Teaching and Research (CULATR) of The University of Hong Kong. To synthesize *Kbtbd7*, *Vangl2*, and *GFP* mRNA for zebrafish embryo injection, 10 μg of pCMV-3Tag-1A-zKbtbd7, HA-Vangl2, and pIRES-hrGFP plasmids was linearized by MluI for in vitro transcription using mMESSAGE mMACHINE T3 Transcription Kit (Thermo Fisher Scientific, AM1348). MOs were synthesized by Gene Tools LLC: Kbtbd7-MO-1 targeted the 5′ untranslated region with a sequence 5′-CTTGCTTTCTCCGTCCAAACGCAAC-3′, and Kbtbd7-MO-2 overlapped the AUG site with a sequence 5′-GAAGCTGTTCACTGAAGCCATGTCG-3′. The Vangl2 MO has been previously described as 5′-GTACTGCGACTCGTTATCCATGTC-3′ (*25*). Standard control oligo targeting a human β-globin intron mutation that causes β-thalassemia was obtained from Gene Tools LLC with a sequence 5′-CCTCTTACCTCAGTTACAATTTATA-3′. Zebrafish embryos at one-cell stage were injected with zKbtbd7 or hrGFP mRNA at 300 pg per embryo, Vangl2 mRNAs at 20 or 60 pg per embryo, Kbtbd7 MO at 2 or 4 ng per embryo, or Vangl2 MO at 0.25, 0.5, or 2 ng per embryo as indicated. Each injection volume was restricted to no more than 4 μl to avoid nonspecific effects. Uninjected, hrGFP mRNA–injected, or control MO–injected embryos were used as the controls. Embryos were imaged 48 hours post fertilization using a Nikon stereo microscope. To check the protein expression, 20 embryos were collected and damaged by pipetting up and down for 15 times. The embryos were allowed to float down to the bottom of the tube to remove deyolking fluid. The embryos were washed three times with PBS to thoroughly remove the yolk. Embryos were lysed in 1× Laemmli sample buffer containing 10% 2-mercaptoethanol and heated at 95°C for 10 min. Overexpressed and endogenous proteins were then analyzed by immunoblotting.

### Gene expression and survival analysis of breast cancer

The expression of *KBTBD7* in normal and breast cancer tissues was analyzed using UALCAN (http://ualcan.path.uab.edu), which is a user friendly and interconnected web resource for analyzing various cancer transcriptome sequencing data (TCGA and MET500) and includes many tumor subtypes. Breast Cancer Gene-Expression Miner (bc-GenExMiner 4.4, http://bcgenex.centregauducheau.fr) was used to evaluate the prognostic association between *KBTBD7* expression level and breast cancer patients’ overall survival and metastatic relapse-free survival.

### Breast cancer xenograft models

All mouse experiments in this study were conducted in compliance with the CULATR of The University of Hong Kong. A total 2 × 10^6^ HCC1806 cells stably expressing firefly luciferase were inoculated into the fourth mammary fat pad of 5- to 7-week-old NOD SCID female mice. The growth of primary tumors was monitored every week. After 4 weeks, the primary tumors were removed surgically under sterile conditions and were weighed to obtain tumor burden. Mice were kept for another 4 weeks to assess the metastasis. Then, all mice were euthanized, and their lungs were collected. The metastasis formation in the lung of the nude mice was assessed by bioluminescent imaging using an IVIS-100 Xenogen in vivo imaging system (PerkinElmer). Before bioluminescence imaging, mice were injected abdominally with 150 mg/kg of d-luciferin (30 mg/ml, Gold Biotechnology) in Dulbecco’s phosphate-buffered saline (D-PBS). Lung metastasis was quantified as the total photon flux (photons per second) and analyzed by Living Image 3.0 software.

### Statistical methods

Statistical data were analyzed using GraphPad Prism 7 (GraphPad Software) with all data presented as means ± SD. Unpaired Student’s *t* test was used to compare two groups, and one-way analysis of variance (ANOVA) was used to compare variance among multiple groups. Differences with *P* < 0.05 were considered statistically significant. The *n* numbers for each group and group numbers are indicated in the figure or figure legends.

## SUPPLEMENTARY MATERIALS

Supplementary material for this article is available at http://advances.sciencemag.org/cgi/content/full/7/20/eabg2099/DC1

## Appendix

View/request a protocol for this paper from *Bio-protocol*.
